# Cellular stress responses to chronic heat shock and shell damage in temperate *Mya truncata*

**DOI:** 10.1007/s12192-018-0910-5

**Published:** 2018-05-12

**Authors:** Victoria A. Sleight, Lloyd S. Peck, Elisabeth A. Dyrynda, Valerie J. Smith, Melody S. Clark

**Affiliations:** 1 Department of Zoology, University of Cambridge, Downing Street, Cambridge, CB2 3EJ UK; 2 British Antarctic Survey, Natural Environment Research Council (NERC), High Cross, Madingley Road, Cambridge, CB3 0ET UK; 3 Centre for Marine Biodiversity & Biotechnology, Institute of Life & Earth Sciences, Heriot-Watt University, Edinburgh, EH14 4AS UK; 4 Scottish Oceans Institute, School of Biology, University of St Andrews, St Andrews, Fife, KY16 8LB UK

**Keywords:** Mollusc, Bivalve, Transcriptomics, Heat shock proteins, Reactive oxygen species, Immunology, Biomineralisation

## Abstract

Acclimation, via phenotypic flexibility, is a potential means for a fast response to climate change. Understanding the molecular mechanisms underpinning phenotypic flexibility can provide a fine-scale cellular understanding of how organisms acclimate. In the last 30 years, *Mya truncata* populations around the UK have faced an average increase in sea surface temperature of 0.7 °C and further warming of between 1.5 and 4 °C, in all marine regions adjacent to the UK, is predicted by the end of the century. Hence, data are required on the ability of *M*. *truncata* to acclimate to physiological stresses, and most notably, chronic increases in temperature. Animals in the present study were exposed to chronic heat-stress for 2 months prior to shell damage and subsequently, only 3, out of 20 damaged individuals, were able to repair their shells within 2 weeks. Differentially expressed genes (between control and damaged animals) were functionally enriched with processes relating to cellular stress, the immune response and biomineralisation. Comparative transcriptomics highlighted genes, and more broadly molecular mechanisms, that are likely to be pivotal in this lack of acclimation. This study demonstrates that discovery-led transcriptomic profiling of animals during stress-response experiments can shed light on the complexity of biological processes and changes within organisms that can be more difficult to detect at higher levels of biological organisation.

**Electronic supplementary material:**

The online version of this article (10.1007/s12192-018-0910-5) contains supplementary material, which is available to authorized users.

## Introduction

Acclimation via phenotypic flexibility is the process in which an individual adjusts to a change in environmental conditions allowing maintained performance and fitness. It has been proposed as the fastest, and potentially most important, response to climate change, particularly for long-lived species with extended generation times and the consequent limited capacity for genetic adaptation (Somero 2012; Bay and Palumbi 2015). Understanding the physiological stress that results from a range of global change-related abiotic and biotic variables, and the associated acclimation mechanisms, is pivotal to deciding an organism’s fate in a given local population (Somero 2012). Many of the previous studies on the acclimation of marine invertebrates have concentrated on responses to warming and used upper lethal temperatures as a measure of successful acclimation to altered conditions (Somero 2010; Peck et al. 2014). More recently, the rise in next-generation sequencing technologies applied to non-model, environmental, species has provided more detailed data on the molecular mechanisms underpinning physiological stress, and hence delivers a more fine-scale cellular understanding of how organisms acclimate, or in some cases fail to acclimate, to changed environmental conditions (Somero 2012; Bay and Palumbi 2015; Moya et al. 2015; Clark et al. 2017).

The subject of this study, *Mya truncata* (Superorder = Imparidentia, Order = Myida, Superfamily = Myoidea, Family = Myidae), is a large sediment-burying bivalve. It is a long-lived species [reported ages up to 40 years (Hewitt and Dale 1984)] that plays an important functional role in bentho-pelagic coupling, sediment stabilisation, and bioturbation (Queiros et al. 2013). In terms of biotic interactions, *M*. *truncata* in temperate habitats are an important food source for many species, such as the sea star *Asterias rubens* and the gastropod *Buccinum undatum* (Himmelman and Dutil 1991; Morissette and Himmelman 2000; Gaymer et al. 2001a; Gaymer et al. 2001b). Despite the large biomass and important functional role of *M*. *truncata* within ecosystems, very little is known about the acclimation potential of this species, particularly at the molecular level.

*Mya truncata* has a boreal-arctic distribution spanning a wide latitudinal range. In marine databases (www.marinespecies.org, www.marlin.ac.uk), it is reported as occurring in the Arctic down to the Bay of Biscay in Europe (~ 79° N to 45° N) (Ballerstedt 2002; Gofas 2004; Oliver et al. 2016), but populations have also been identified in the Mediterranean: Monaco and the southern (Atlantic) shores of Spain [specimens in the Muséum National d’Histoire Naturelle, Paris (E. Harper, pers. comm., Checa, (1993)]. Ecological observations of this species have concentrated on the Southern North Sea as part of a long-term benthic macrofauna monitoring programme (Amaro et al. 2005; Witbaard et al. 2005). These studies have shown that numbers of *M*. *truncata* are drastically reduced with decreasing latitude, with up to 100-fold less individuals at latitudes around the Frisian front [< 1 individual per m^2^ (Amaro et al. 2003)] compared to Arctic latitudes [> 100 individuals per m^2^ (Welch et al. 1992)]. In addition, *M*. *truncata* frequently failed to recruit at the Frisian Front. From 1987 to 2001, the species was absent from survey records at the Frisian Front (Amaro et al. 2003), and individuals had low numbers of ripe oocytes indicating reduced reproductive fitness (Amaro et al. 2005). Based on these data, it would appear that distributions of *M*. *truncata* are impacted by temperature, but that the species, in general, has a moderate window of physiological capability. Populations in the Arctic (circa. 79° N) can experience 9 months at sub-zero temperatures with 1.5 m of ice cover (Hewitt and Dale 1984), whereas temperate populations experience no ice cover and summer temperatures in excess of 15 °C (in the Mediterranean for example). To date however, apart from the environmental information described above, there are no experimental data on either upper lethal temperatures or the thermal tolerances of *M*. *truncata*.

All across *M*. *truncata*’*s* boreal-arctic distribution populations are experiencing warming as a result of anthropogenic climate change. In the last 30 years, *M*. *truncata* populations around the UK have faced an increase in sea surface temperature of 0.7 °C (Frost et al. 2016). Using a medium emissions scenario, the UK Climate Projections 2009 (UKCP09) predict further future warming, in all marine regions adjacent to the UK, of between 1.5 and 4 °C. Given the previous surveys suggesting a decrease in population densities with latitude, data are required on the ability of *M*. *truncata* to tolerate multiple physiological stresses, and most notably, chronic increases in temperature, in order to predict the likely ecological fate of this species in the future.

The aim of the present study was to evaluate the ability of *Mya truncata* to perform damage-repair under a future climate change scenario. Temperate subtidal *M*. *truncata* were acclimated to chronic warming (2 months at 16 ± 1 °C; 1 °C above current maxima) which was followed by artificially induced shell damage. This experiment represented the multi-faceted nature of survival in a changing world with warming and the ecological interaction of simulated predation. Transcriptomic profiling of individual animals over a 2-week time course after shell damage was used to provide insight into the molecular mechanisms underpinning responses to these two combined physiological stresses and the cellular impacts associated with life at a higher temperature.

## Methods

### Animal collection, habitat parameters and animal husbandry

**Fig. 1:**
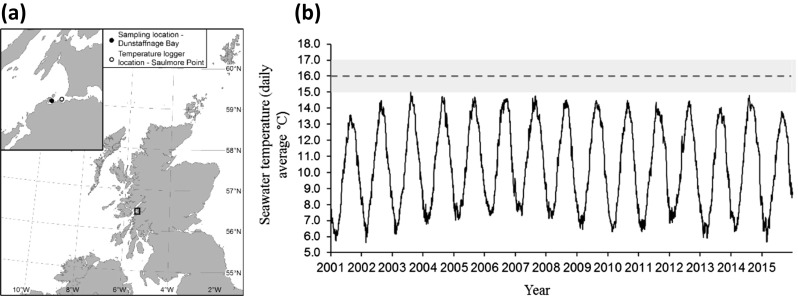
**a** Sampling and temperature logger locations. **b** Daily average seawater temperature (*n* = 240 readings per day) from January 2001 to December 2015. Temperatures recorded by a logger submerged to 10 m depth at Saulmore Point (mouth of Loch Etive, opposite Dunstaffnage Bay [056° 27′ N, 005° 24′ W], **a**). Data provided by Martin Sayer from the UK National Facility for Scientific Diving. Dashed line and grey region represents experimental holding temperatures (16 ± 1 °C)

Specimens of *Mya truncata* were hand-collected by SCUBA divers at the UK National Facility for Scientific Diving from Dunstaffnage Bay, North West Scotland (56° 27′ 06.5′′ N 5° 26′ 02.2′′ W) between April and May 2016 (Fig. 1a). All specimens were sexually mature adults (mean shell length = 65 ± 1 mm S.E). All collections were from a depth of 10–15 m. Data on habitat temperatures were obtained from Martin Sayer (UK National Facility for Scientific Diving). A temperature logger, continuously running on a mooring, on the edge of Dunstaffnage Bay at 10 m depth (Saulmore Point, 56° 27′ 14.6′′ N 5° 24′ 48.6′′ W) was used to measure temperature every 10 min—which was then converted into a daily average—from January 2000 to October 2016 (Fig. 1). These data were used to select an appropriate elevated temperature (16 °C) for the heat-stress experiment. Heat-stress in this study was defined as 1 °C higher than any ambient condition animals from the sampled population would experience in their natural habitat (Fig. 1b). In addition, an increase in temperature of just 1 °C is a very conservative prediction of warming in the region sampled (using a medium emissions scenario, the UK Climate Projections 2009 (UKCP09) predict warming, in all marine regions adjacent to the UK, of between 1.5 and 4 °C) and allows sub-lethal effects to be measured (such as acclimation mechanisms).

After collection, clams were immediately transferred to the Scottish Association of Marine Science (SAMS) flow-through aquarium (salinity 35–38 ppt) where they were randomly distributed into three tanks. An elastic band was placed around each specimen to prevent gaping and animals were laid on top of approximately 20 cm of sand, which they could bury into. The animals were maintained under a 12:12 h simulated light:dark cycle and fed a pre-mixed microalgal blend twice weekly throughout acclimation and experimentation (Shellfish Diet, Varicon Aqua solution Ltd., UK).

### Experimental design

**Fig. 2:**
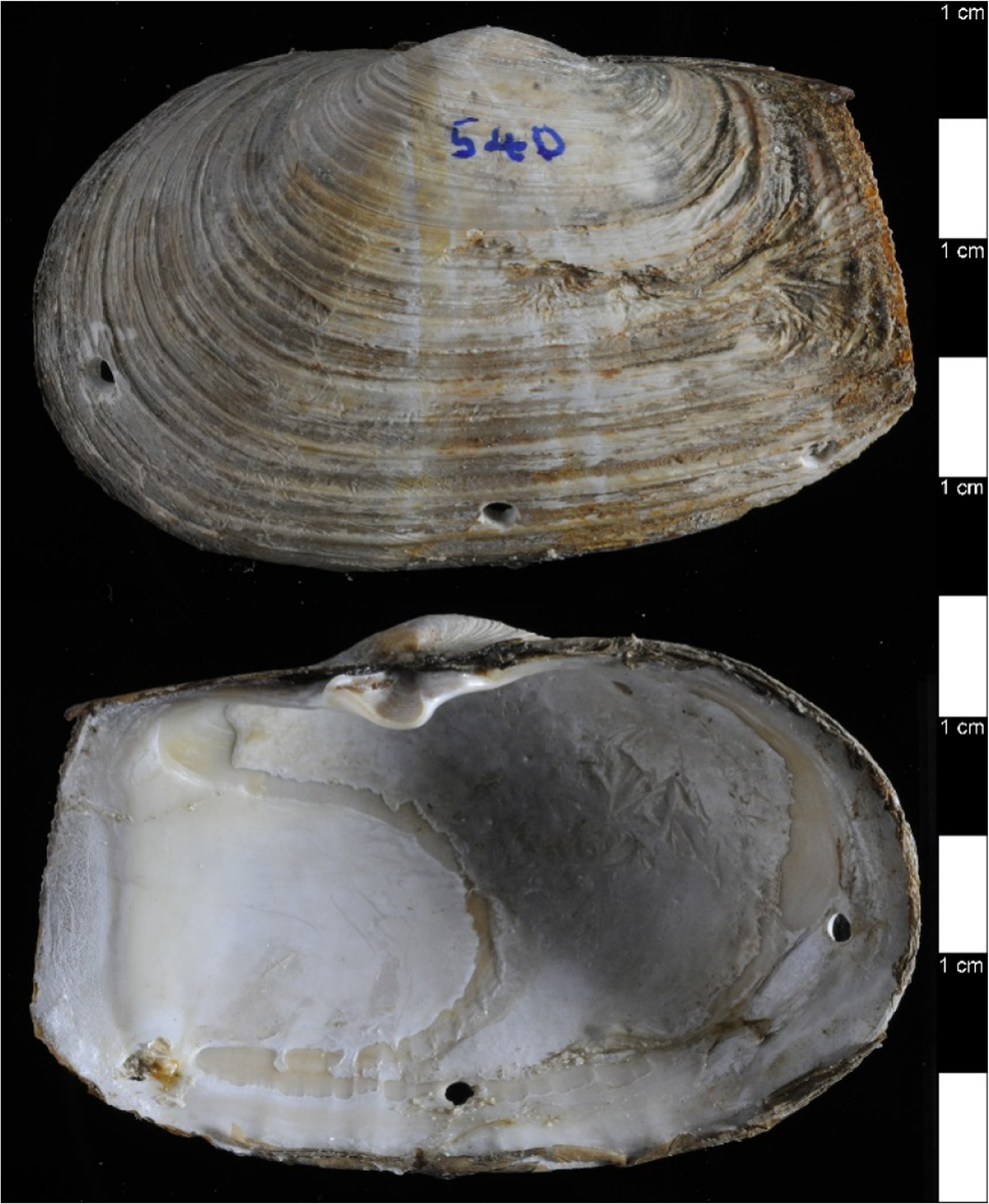
Demonstration of shell damage treatment. Top = outside view of *M*. *truncata* shell, bottom = inside view of same shell. 1 cm scale down the right-hand side

The experiment entailed an initial chronic, long-term exposure to elevated temperature followed by the response to shell-damage, which was assessed at the molecular level over a 2-week time course. For the long-term heat exposure, 40 individuals were kept at 16 ± 1 °C (1 °C higher than they would experience in their natural habitat, as per the habitat temperature data, Fig. 1b) for 2 months. Twenty of these heat-exposed animals were then subjected to shell damage. Shells were damaged by drilling three holes (approximately 2.5 mm in diameter) through the shell, just inside the pallial line close to the ventral edge, within reach of the mantle margin, using a 10.8 V Lithium-Ion Dremel cordless modelling drill (at a speed of 5000–35,000 rpm) fitted with a round-tipped bit to minimise any trauma to the underlying soft tissue as in Sleight et al. (2015). To inflict damage, animals were lifted out of the water, placed on crushed ice on a bench in the aquarium where their shells were drilled and returned immediately to the aquarium tank. Control animals were similarly removed from the tank and placed on ice, before returning to the tank, to control for handling stress. In order to assess repair after damage, shells were removed from the animal and examined for evidence of repair on both internal and external surfaces (Fig. 2). The remaining 20 animals were left as undamaged for controls. All animals were then maintained at 16 °C before sampling of both control and damage individuals over a time course at 1, 3, 5, 7, and 14 days post-treatment (*n* = 4 per treatment, per time point).

### Haemocyte counts and shell damage-repair

**Fig. 3:**
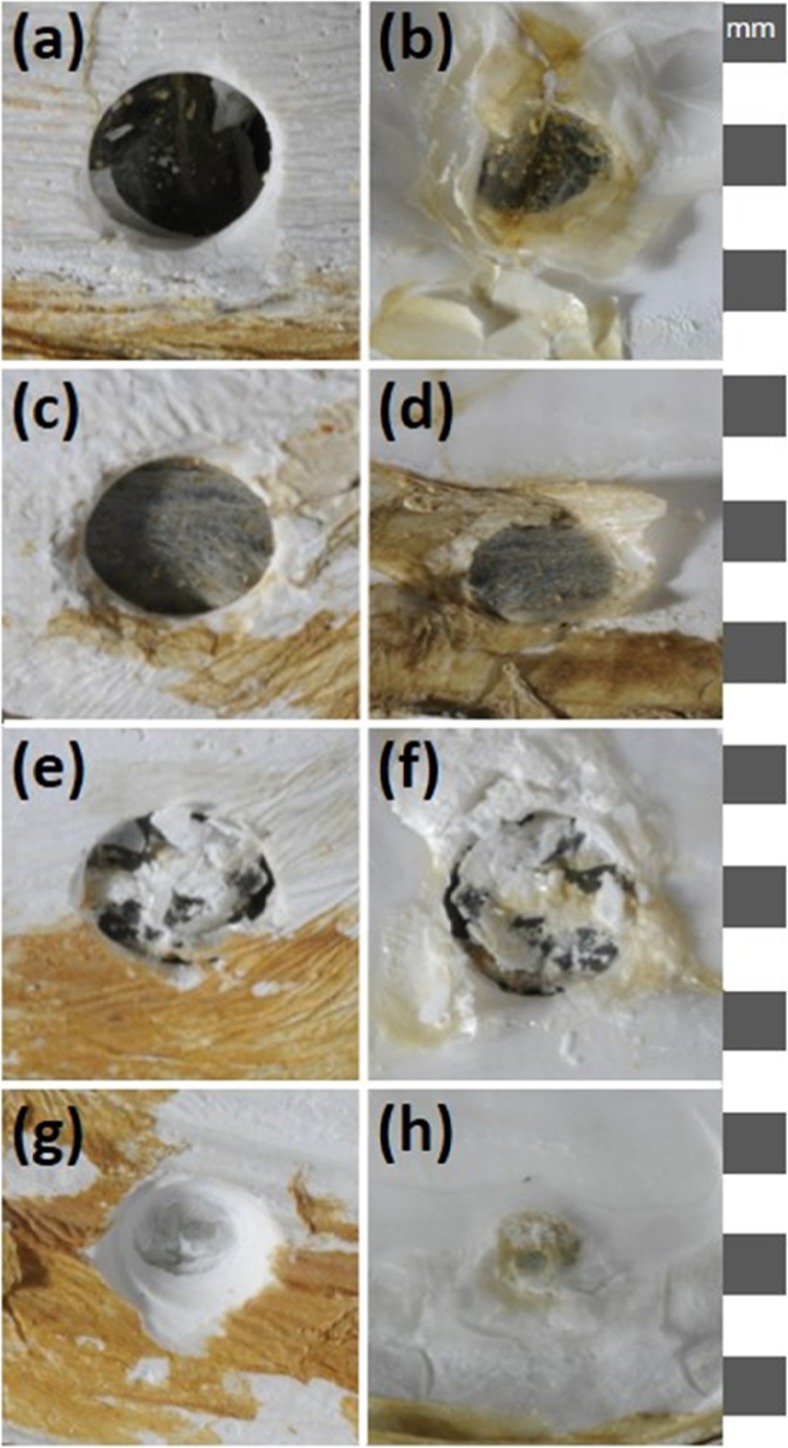
Photographs of shell repair categories. **a** Category 1, thin clear film viewed from outside of shell. **b** Category 1 viewed from inside of shell. **c** Category 2, translucent brown proteinacious film viewed from outside of shell. **d** Category 2, viewed from inside of shell. **e** Category 3, partially calcified brown film viewed from outside of shell. **f** Category 3 viewed from inside of shell. **g** Category 4, fully calcified layer, viewed from outside of shell. **h** Category 4 viewed from inside of shell. 1 mm scale down the right-hand side. N.B. For illustrative purposes only, these are not *M*. *truncata* shells from this study as animals in this study did not heal past category 3

At each time point of the experiment (see above), animals were removed from each tank and were photographed to assess the degree of repair in the damaged shells (*n* = 4). The stage of repair was assigned to one of four qualitative repair categories (Fig. 3). Given the shell damage method caused a breach in the external barrier of the animal, it was likely to cause an immune challenge, and hence it was predicted the immune system would be involved in the response. As a proxy for immune activity, haemolymph (fluid equivalent to blood in most invertebrates [250 μL]) was extracted from the adductor muscle of each individual into a syringe containing 250 μL of 4% formaldehyde in 0.22 μm filtered sterile seawater and fitted with a 25 gauge needle. The total number of haemocytes (invertebrate immune cells) per millilitre (THC) of haemolymph was counted using an improved Neubauer haemocytometer under a light microscope. Mantle tissue samples were then taken across the three mantle folds from a region under the middle hole as described in Sleight et al. (2015).

### Sequencing and bioinformatics

Total RNA was extracted from each mantle tissue sample on ice using Tri-Reagent (Bioline, UK), and purified using RNeasy columns including a DNase treatment (QIAGEN, UK), all performed according to manufacturer’s instructions. The RNA samples were analysed for concentration and quality by spectrophotometer (NanoDrop, ND-1000) and tape station analyses (Agilent 2200 TapeStation). The best three RNA samples from the four animals extracted at each time point was sent to the sequencing centre. cDNA libraries were made for each individual (*n* = 30; 15 treated animals from 5 time points (*n* = 3 at each time point), with a corresponding number of controls) and library preparation was conducted by the Earlham Institute, Norwich, UK (formerly The Genome Analysis Centre). Stranded libraries were prepared using the NEXTflex™ Rapid Illumina Directional RNA-Seq Library Prep Kit and sequencing was carried out on a Hi-Seq 2000, generating 100 base paired-end reads. Reads went through an initial quality control process conducted by the Earlham Institute that removed Illumina adaptor sequences and ribosomal RNA reads. Reads were then further cleaned for quality (Phred score 30) and minimum read length (80 bp) using the eautils (v1.1.2) tool fastq-mcf (https://github.com/ExpressionAnalysis/ea-utils/blob/wiki/FastqMcf.md). The cleaned reads were normalised using Trinity’s (v2.2.0) In silico Read Normalisation tool (Haas et al. 2013), with default parameters. The left and right reads for each library were normalised; all of the left and right reads were then concatenated and the resulting file was normalised a second time with default parameters. The concatenated, normalised reads were de novo assembled using Trinity (v2.2.0) with default parameters (Grabherr et al. 2011). For a quantitative assessment of orthology completeness, the assembled transcriptome was subject to BUSCO analysis (Benchmarking Universal Single-Copy Orthologs, v3) using the entire Metazoa dataset (*metazo*_*odb9* downloaded January 2018).

Transcript abundance was estimated by alignment-based quantification using Trinity (v2.2.0) utilities (Grabherr et al. 2011; Haas et al. 2013). Transcripts were aligned to the de novo transcriptome using bowtie with default parameters and transcript abundance estimates were calculated using RNA-Seq by Expectation-Maximisation (RSEM). The gene-level abundance estimates (raw counts) for each of the libraries were constructed into a matrix for downstream expression analyses (using the Trinity abundance_estimates_to_matrix.pl script). Transcript abundance estimation was quality checked using the Trinity Perl-to-R ‘PtR’ toolkit and principle component analysis was used to check for batch effect and outliers (Haas et al. 2013). Outliers were identified (one sample from the 1-day time point and two samples from the 5-day time point) and removed, which significantly unbalanced the experimental design and hence the 5-day time point could not be included in downstream analysis (Supplementary Information, Fig. S1, for more details about outlier were removal). Differentially expressed genes, between control and damaged animals at each time point, were identified using the Bioconductor (v3.4) edgeR package in R (v3.1.1) with a false discovery rate (FDR) of 5% and a twofold change cut-off (Robinson et al. 2010; McCarthy et al. 2012). In the same analysis, genes that showed significant temporal changes in response to damage, termed here, time-dependant damage-response genes, were identified. The full R script for all downstream analyses and the count data matrix are available (Supplementary Files S1 and S2).

The longest isoform of each gene was extracted from the transcriptome for annotation as in An et al. (2014), using the Trinity utility script ‘get_longest_isoform_seq_per’. The longest isoforms of each gene were compared to a local NCBI non-redundant (NR) database (updated 01 June 2016) using Basic Local Alignment Search Tool (blastx, cut-off <1e^−10^) to search for sequence similarity and putative gene annotation (Altschul et al. 1990).

To aid interpretation of the hundreds of up- and down-regulated genes, and to provide a visual qualitative assessment of the different biological processes at each time point, STRING (v10.0) was used to produce protein-protein interaction networks (Franceschini et al. 2013; Szklarczyk et al. 2015). All of the differentially expressed genes at each time point were compared to a local UniProt/SwissProt human database (updated 05 October 2016) using Basic Local Alignment Search Tool (blastx, cut-off <1e^−10^, Table S1) and these identifiers used as input into STRING. STRING only accepts identifiers from UniProt/SwissProt accession numbers and human data are the most abundant and best annotated dataset available, providing the most comprehensive coverage compared with other model species, in spite of the evolutionary distance (Clark, pers. comm.). Protein interaction scores in STRING were set to ‘high confidence 0.7’, only the query proteins were included in the network and all non-interacting proteins were removed from the analysis. Functional enrichment analysis of each network was performed using the whole human genome as a statistical background (as above, STRING uses human annotations and therefore compared to human genome as a background). In addition to the STRING protein-protein interactions, all annotations (blastx against NR and UniProt/SwissProt human) of the differentially expressed genes were manually interrogated and assigned a putative function.

### Availability of data and material

All raw sequence data has been submitted to NCBI SRA and is publically available (accession number: SRP115712). The mantle transcriptome assembly is available upon request and the code and data matrix used in downstream analysis are available in the electronic supplementary material.

## Results

**Fig. 4:**
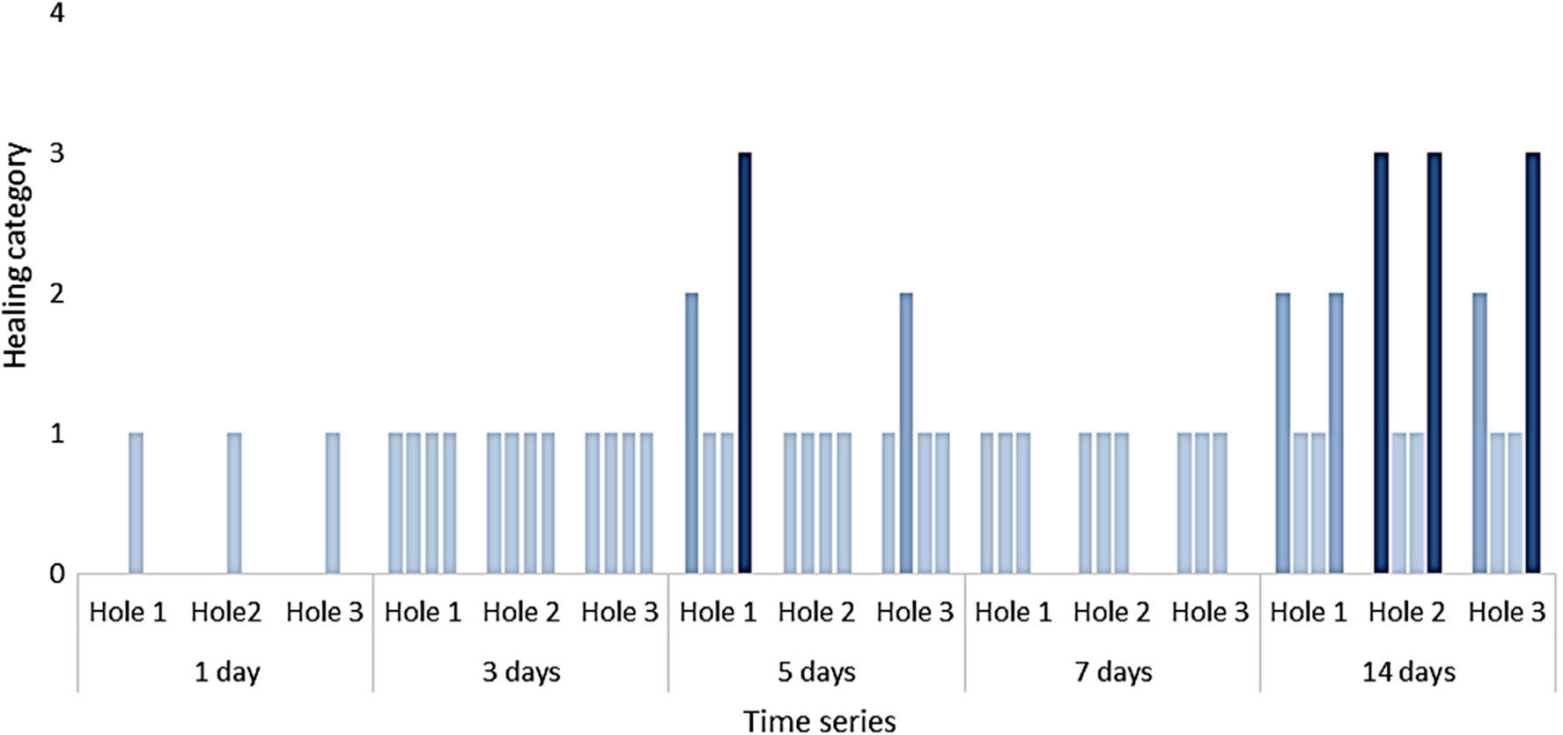
Shell repair observed at each hole at each time point for each damaged individual (*n* = 4 per time point). Healing categories as per Fig. 3

Only 3, out of 20 damaged individuals, reached healing category 3 ‘holes covered by a brown film which shows signs of partial calcification’ after 14 days (Figs. 3 and 4). There was no difference in THC between control and damaged individuals at any time point (Supplementary Information, Fig. S2).

All of the RNA libraries (*n* = 30) were cleaned, normalised and assembled into a de novo transcriptome (Table 1). The assembly and downstream transcript abundance estimates were quality-checked (Table 1 and Supplementary Information, Fig. S1) and deemed suitable for further analysis, 16% of Trinity genes were assigned putative annotation using blastx sequence similarity searching against the NR database (below an *e* value of 1e^−10^).

**Table 1:** Assembly statistics for *M*. *truncata* mantle de novo transcriptome

Reads	
Raw reads	316,857,250
Clean reads (q30, l80)	287,520,918
Normalised reads (K25, C50, pctSD200)	52,205,278
Assembly	
Total trinity transcripts	684,686
Total trinity genes	438,210
GC (%)	38.9
Statistics based on longest isoform per gene	
N50 (bp)	497
Median length (bp)	297
Mean average length (bp)	455
BUSCO against whole metazoan gene set (*n* = 978)	
Total completeness (%)	97.3
Complete and single-copy BUSCOs (%)	64
Complete and duplicated BUSCOs (%)	33.3
Fragmented BUSCOs (%)	2.5
Missing BUSCOs (%)	0.2

**Fig. 5:**
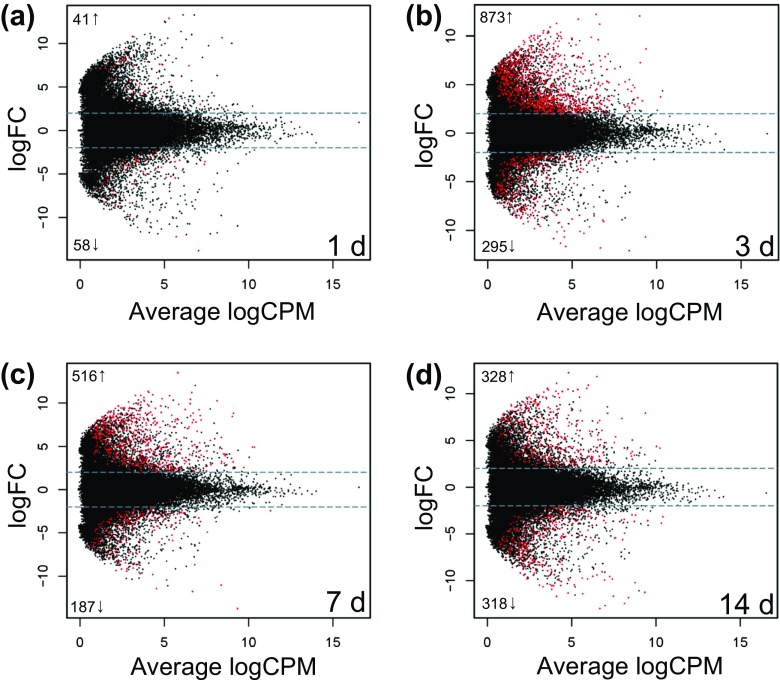
Smear plots of the up- and down-regulated genes between damaged and control animals at each time point **a** 1 day after damage, **b** 3 days after damage, **c** 7 days after damage and **d** 14 days after damage. Grey dashed lines indicate log 2-fold cutoff and genes which were found to be significantly different by edgeR are indicated in red, the number of significantly up- and down-regulated genes indicated on the left of the smear

Differential gene expression analysis using edgeR revealed that 1 day after shell damage, 41 (3 annotated) genes were up-regulated and 58 (33 annotated) genes were down-regulated in damaged compared to control animals; 3 days after shell damage, 873 (135 annotated) genes were up-regulated and 295 (103 annotated) genes were down-regulated; 7 days after shell-damage, 516 (81 annotated) genes were up-regulated and 187 (58 annotated) genes were down-regulated; and finally 14 days after shell damage, 328 (93 annotated) genes were up-regulated and 318 (62 annotated) genes were down-regulated (Fig. 5 and Supplementary File S3).

Protein-protein interaction networks were constructed out of the annotated, differentially expressed genes between damaged and control animals at each time point. The networks were used to provide a visual qualitative assessment of the different biological processes at each sample time. One day after damage treatment, transcription and protein transport were the differential processes between damage and control animals. Three days after damage, transcripts associated with RNA processing, cell cycle regulation, wound healing, immunity, ion transport, biomineralisation, stress responses, protein turnover, lipid metabolism, amino acid metabolism and muscle contraction were differentially expressed. Seven days after damage, differences in transcriptomic profiles between experimental and control animals were those involved in RNA processing, cell cycle regulation, stress response, amino acid metabolism and apoptosis. Finally, 14 days after damage, the differences in the transcripts were those governing: cell cycle regulation, stress responses, lipid metabolism, apoptosis, immunity, cell differentiation and development. Each network was analysed for functional enrichment (FDR < 0.05) using GO terms, against the whole genome background. One day after damage, there were no functional enrichments whereas, after 3 days, 12 biological processes (including immune and stress responses) and 9 cellular processes (mainly extracellular and vesicle associated processes) were enriched. Subsequently, 7 days after damage, 14 biological processes (including biomineralisation and immune processes) and 8 cellular processes (again mainly extracellular and vesicle related processes) were enriched. By 14 days post-treatment however, transcripts for only two biological processes (stress and immune) and no cellular processes were enriched (Table 2).

**Fig. 6:**
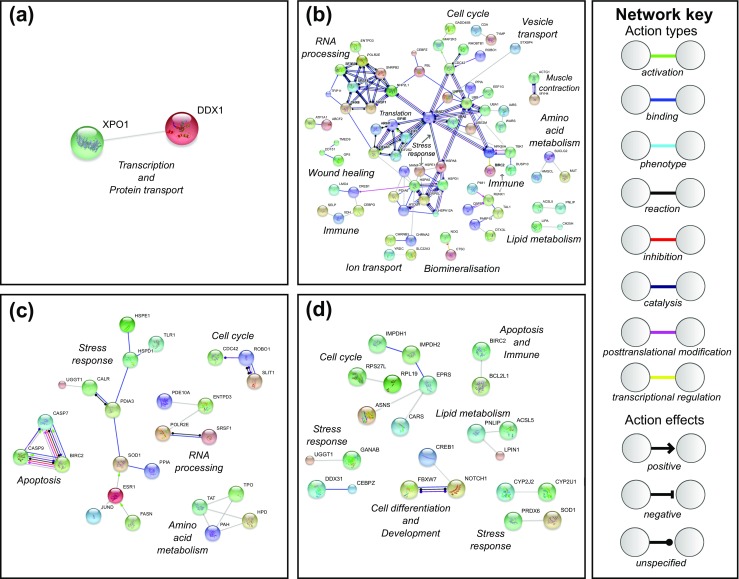
STRING Database predicted protein-protein interactions built from differentially expressed genes between control and damaged treatments. **a** 1 day after damage, **b** 3 days after damage, **c** 7 days after damage and **d** 14 days after damage. Line colour and type depict molecular actions and effects as per the network key and manually added italicised labels indicate most likely associated biological processes or pathways. Please see Table S1 in supplementary information for all abbreviations

**Table 2:** Functional enrichment of each network in Fig. 6 as per gene ontology (GO) using the whole genome as a statistical background

Pathway ID	Pathway description	Observed gene count	False discovery rate
Time point 1: 1 day			
Biological process (GO)			
None			
Cellular component (GO)			
None			
Time point 2: 3 days			
Biological process (GO)			
GO.0008152	Metabolic process	111	0.0154
GO.0006807	Nitrogen compound metabolic process	73	0.0212
GO.0034138	Toll-like receptor 3 signalling pathway	7	0.0212
GO.0035666	TRIF-dependent toll-like receptor signalling pathway	7	0.0212
GO.0044238	Primary metabolic process	100	0.0212
GO.1901564	Organonitrogen compound metabolic process	32	0.0212
GO.0002224	Toll-like receptor signalling pathway	8	0.0246
GO.0071704	Organic substance metabolic process	100	0.0269
GO.0006950	Response to stress	51	0.0299
GO.0002221	Pattern recognition receptor signalling pathway	8	0.0404
GO.0010033	Response to organic substance	40	0.0404
GO.0034142	Toll-like receptor 4 signalling pathway	7	0.0404
Cellular process (GO)			
GO.0005576	Extracellular region	73	8.21E-06
GO.0044421	Extracellular region part	62	8.63E-05
GO.0031982	Vesicle	58	0.000291
GO.0031988	Membrane-bounded vesicle	57	0.000291
GO.0070062	Extracellular exosome	49	0.000291
GO.0034663	Endoplasmic reticulum chaperone complex	3	0.0227
GO.0016222	Procollagen-proline 4-dioxygenase complex	2	0.0385
GO.0044444	Cytoplasmic part	86	0.0385
GO.0042470	Melanosome	6	0.0421
Time point 3: 7days			
Biological process (GO)			
GO.0001649	Osteoblast differentiation	8	0.00153
GO.0044710	Single-organism metabolic process	40	0.00584
GO.0001503	Ossification	9	0.0122
GO.0008152	Metabolic process	65	0.0122
GO.0044712	Single-organism catabolic process	16	0.0122
GO.0006559	L-phenylalanine catabolic process	3	0.0222
GO.0006570	Tyrosine metabolic process	3	0.0222
GO.0071704	Organic substance metabolic process	60	0.0222
GO.0048731	System development	33	0.0292
GO.0031638	Zymogen activation	6	0.0318
GO.0048856	Anatomical structure development	36	0.0318
GO.0048513	Organ development	27	0.038
GO.0051604	Protein maturation	8	0.038
GO.0030099	Myeloid cell differentiation	7	0.0418
Cellular process (GO)			
GO.0005576	Extracellular region	41	0.000855
GO.0005615	Extracellular space	20	0.000855
GO.0044421	Extracellular region part	38	0.000855
GO.0070062	Extracellular exosome	31	0.000855
GO.0043227	Membrane-bounded organelle	73	0.00652
GO.0031982	Vesicle	33	0.0112
GO.0031988	Membrane-bounded vesicle	32	0.0139
GO.0043226	Organelle	73	0.0367
Time point 4: 14days			
Biological process (GO)			
GO.0042738	Exogenous drug catabolic process	4	0.0393
GO.0044710	Single-organism metabolic process	42	0.0393
Cellular process (GO)			
None			

In addition to investigating differential expression within each time point, genes that showed significant temporal changes across time in response to damage (termed here, time-dependant damage-response genes) were also identified. The top 50 most significantly different (smallest FDRs), annotated genes, were selected from 6103 identified time-dependant damage-response genes (Supplementary File S4). Genes that were increasingly up-regulated over the time series (in damaged compared to control animals) included putative candidates in biomineralisation such as *collagen*, *calumenin*, *matrilin* and *pif*; the immune response such as *calumenin* (n.b. multi-functional), *theromacin* and *L*-*rhamnose*-*binding lectin*, and also the stress response, such as the detoxification genes *cytochrome P450 3A24* and the chaperone *heat shock protein 60*.

## Discussion

The present study shows that *M*. *truncata* displays evidence of a lack of acclimation under a predicted end-of-century climate change scenario with a consequential inability to successfully repair shell damage. Transcriptomic profiling revealed consistent evidence for stress responses to be the key players in the molecular mechanisms underlying this lack of acclimation and ability to repair shell damage. At each time point, genes were differentially expressed between control and damaged animals (Fig. 5) and, those differentially expressed genes were functionally enriched with known cellular stress response candidates (Table 2, Fig. 6, Supplementary Information, Supplementary file 1). In addition, genes that showed the most significant temporal changes in expression between damaged and control animals across the time series, i.e. time-dependant damage-response genes, also included classic stress response genes such as the detoxification genes *cytochrome P450 3A24* and the chaperone *heat shock protein 60* (Supplementary file 4).

Previous work has shown that *M*. *truncata* frequently have low fecundity and a failure to recruit at the Frisian Front (a region of relatively similar latitude and temperature range to the specimens used in the present study). Furthermore, previous work has also shown that molluscs (*Margaritifera margaritifera)* living at high latitudes with a longer lifespans have better wound and shell-healing capacity than those living at lower latitudes (Ziuganov et al. 2000). The difference in repair capacity between high and low latitude populations of *M*. *margaritifera* was hypothesised to be due to the reduced energy expenditure for growth, and therefore greater energy availability for shell and tissue regeneration to sustain homeostasis. Interestingly, neither the Frisian Front nor Dunstaffnage Bay are close to the southern range boundaries of this species (E. Harper, pers.comm., Checa 1993) and therefore do not represent populations living in the warmest sea temperatures this species is known to inhabit. *M*. *truncata* are broadcast spawners with planktonic larvae (Amaro et al. 2003), but to date, very little is known about the population genetics of this species or the dispersal capabilities of the larvae. The most likely explanation therefore is that there is a lack of connectivity between these northern populations and those in the Mediterranean, which are more adapted to warmer waters. Unpublished population genetics data on a sister species, *Mya arenaria*, support this explanation (specifically, distinct populations in Scotland, northern Europe, Mediterranean and Spain/Portugal; M. De Noia & D. Vendrami, pers. comm.). Previous studies have shown that vulnerability to warming in a species is context dependent and does not follow a strict latitudinal pattern (Sagarin and Somero 2006; Osovitz and Hofmann 2007). Hence, combining the experimental data presented in the present study, with the recent trend of oceanic warming around the UK of 0.7 °C in the last 30 years (Frost et al. 2016), suggests that some temperate *M*. *truncata* populations are being pushed past their viable thermal tolerance limit and possibly impacting the availability of cellular energy levels, reflected by an inability to repair shell damage.

The transcriptional profiling results show activation of cellular stress responses. Functional enrichment analysis of the differentially expressed genes between control and damaged animals demonstrated that cellular stress responses were enriched at 3, 7 and 14 days post-damage. These responses could be split into two broad categories, the classical heat shock response and antioxidant production.

Heat shock proteins (hsps) are involved in protein folding and chaperoning. They are either constitutively expressed as part of normal cellular function or induced in response to stress (Hartl 1996) as they recognise and repair mis-folded proteins and also target degraded proteins by regulating their removal (Fink 1999). In an earlier study, a mantle transcriptome was produced from animals sampled within days of collection and revealed that two *hsp* genes were highly expressed, *heat shock protein 90*-*alpha 1* and *heat shock protein 70* (Sleight et al. 2016). It was suggested that one possible explanation for the high background expression of *hsps*, particularly the inducible *hsp70*, was that the animals were exhibiting low levels of thermal stress. In the present study, multiple *hsps* were significantly up-regulated in damaged animals, compared to undamaged controls, at the 3-, 7- and 14-day time point (Supplementary File 3) and in addition, two *hsps* were detected in the top 50 most significant time-dependant damage-repair genes (Fig. 6 and Supplementary File 4). These up-regulated *hsps* included two members of the *hsp70* superfamily, one of which was clearly *hsp7012A*, an *hsp60* and two small heat shock proteins (Table 3). Detailed comparison of the other HSP70 family member described in this study with the *hsp70* identified in Sleight et al. (2016) showed clear differences with 71.8% sequence identity at the amino acid level (Supplementary Information, Fig. S3). The sequences of both transcripts included the classic motifs for members of the HSP70 superfamily, in particular the R-A-[RK]-F-E-[ED]-[LM] motif characteristic of HSP70, HSC70 and GRP78 proteins (Supplementary Information, Fig. S3). BLAST sequence similarity searching discounted the likelihood of either sequence as being GRP78 and therefore they were both designated as *hsp70* (Table 3)*.* It is likely that since the *hsp70* in Sleight et al. (2016) was identified under ‘normal’ environmental conditions, this is putatively the constitutive form (*hsc70*), whilst the transcript identified in the present study is the inducible form, (*hsp70*) up-regulated in response to chronic heat shock and shell damage, future functional analyses will be required to confirm this.

**Table 3:** BLAST sequence similarity searching results for the up-regulated heat shock proteins identified in both Sleight et al. (2016) and the present study

Contig I.D.	Heat shock protein designation	*e* value	Species name	Accession number
Sleight et al. (2016)				
Contig00187	*hsp90*	0.0	*Corbicula fluminea*	AMM04544
Contig00268	*hsp70*	0.0	*Corbicula fluminea*	AHY03302
The present study				
DN151480_c2_g3	*hsp7012A*	3e-145	*Mizuhopecten yessoensis*	OWF47124
DN157452_c1_g1	*hsp60*	0.0	*Ruditapes philippinarum*	AMY16439
DN156808_c2_g1	*hsp70*	0.0	*Mytilus galloprovincialis*	CAH04107
DN154162_c3_g3	*hsp30*	5e-37	*Crassostrea gigas*	XP_022323008
DN133517_c0_g1	*hsp10*	7e-45	*Crassostrea gigas*	XP_022307133

The induction of heat shock protein genes in response to various types of stress has been documented in many marine invertebrates and although these can be viewed as a generic response, the up-regulation of particular genes is often context-dependent (Schill et al. 2003; Chang 2005; Clark and Peck 2009; Huang et al. 2013). The ability to predict the vulnerability of a species to environmental change is best achieved at the molecular level, as sub-lethal effects across a range of functions can be more easily detected and evaluated (Clark and Peck 2009). In addition to identifying up-regulated *hsps* within and across time points in the present study, protein-protein interaction networks indicated that up-regulated *hsps* could be acting as molecular hubs, interacting with a variety of functional pathways including immune response, ion transport and ubiquitination/protein turnover (Fig. 6) (Csermely 2004; Korcsmaros et al. 2007). More specifically, protein-protein interactions within functional pathways revealed that up-regulated *hsps* could be regulating stress-induced cell death. The *hsp* cluster in the 3-day post-damage protein-protein interaction network included three connections to a *mesencephalic astrocyte*-*derived neurotrophic factor* gene, which has been shown to mediate stress-induced cell death (Yang et al. 2014). This is in line with experiments showing that failure to repair stress-induced unfolded proteins and to remove aggregated proteins leads to the activation of apoptotic cell death pathways (Powell et al. 2005). The present study provides further evidence that *hsps* underpin cellular responses to stress and their high expression in damaged animals, who were unable to repair shell damage after chronic exposure to elevated temperature (+ 1 °C), points to a molecular mechanism which is likely to be critically important in stress-tolerance this species.

The other major functional category of up-regulated genes was antioxidant activity. Oxidative stress can be described as an imbalance between the production of reactive oxygen species (ROS) and the ability of an organism to counteract or detoxify their harmful effects via antioxidants (Jones and Go 2010). Transcriptomic profiling revealed that oxidative stress was most prominent 14 days after damage where antioxidant genes such as *glutathione synthase*-*like* (Noctor and Foyer 1998; Wu et al. 2004) and *spermine oxidase*-*like* (Bouchereau et al. 1999) were significantly up-regulated (Supplementary File 3). In addition, *cytochrome P450*—one of many genes which encodes an enzyme that most commonly catalyses a monooxygenase reaction to metabolise xenobiotics—was detected as one of the 50 most significant time-dependent damage-response genes (Supplementary file 4). As well as generating potent ROS during xenobiotic metabolism, cytochrome P450s have also been shown to have protective effects against oxidative stress, and therefore, overall, must contribute to an organism’s oxidative balance (Caro and Cederbaum 2004; Lingappan et al. 2014). Accurately assessing oxidative balance in an organism, and interpreting what different oxidative states mean for ecology and evolution, is an incredibly complex problem (Cohen et al. 2010; Haussmann and Marchetto 2010). The combination of chaperone production and the response to ROS, as seen here however, is indicative of a failing physiological state.

The differentially expressed genes between control and damaged animals were functionally enriched with immune processes both 3 and 7 days post-damage (Table 2). Like all protostome invertebrates, molluscs rely entirely on the innate defences for host protection. These defences comprise two main parts: recognition and direct effector mechanisms. Regarding the recognition phase, 3 days post-damage, differentially expressed genes were functionally enriched with five receptor-based GO terms (for example, Toll-like receptor 3 signalling pathway, FDR = 0.02). More detailed interrogation of the gene sequences that were significantly up-regulated in the damaged animals includes one encoding a fibrinogen-like domain (Supplementary File S3). Rather than this sequence being the full fibrinogen molecule itself (which is primarily involved on clotting in vertebrates), it is more likely that it is part of a gene that encodes one of the hypervariable family of fibrinogen-related domain (FReD) proteins (Hanington and Zhang 2011). FReDs are pathogen recognition receptors that are responsible for binding non-self-moieties. The presence of FReDs in the differentially expressed genes is therefore highly indicative of an immune response by *M*. *truncata.* Other genes that point to recognition events in the present study include those that encode for various lectins (most notably ficolin) as well as C1q and leucine-rich repeat-containing proteins (LLRs)*.* Lectins are glycoproteins that have two sugar-specific binding sites. Their binding properties facilitate recognition and agglutination of non-self-materials with ficolins binding *n*-acetyl glucosamine, a molecule present in the cell walls of bacteria. *C1q* is expressed in a wide variety of tissues, including the bivalve mantle (Gerdol et al. 2015) and, although it is regarded as a pattern recognition receptor in these animals, in mammals it is part of the classical complement system that binds antibody. Bivalves do not express immunoglobulin antibodies but their C1q protein binds lipopolysaccharide, peptidoglycan, and β-glucan (Zhang et al. 2008; Wang et al. 2012), all of which are pathogen-associated molecular patterns (PAMPs) on microbial surfaces. Lastly, among the differentially expressed genes found in *M*. *truncata* that represent immune recognition are the LLRs. In many species, including vertebrates, proteins with leucine-rich repeats are well represented as hypervariable receptors and are best known as Toll or Toll-like molecules (Zheng et al. 2005) and the variable lymphocyte receptors of lamprey (Pancer et al. 2004). All of these genes, except *C1q*, were up-regulated at day 3 of the study, which fits with the roles of the encoded proteins being part of an early immune response. F*ibrinogen*, *ficolin* and *LLR* genes were still highly expressed at 7 days, and *ficolin*, *C1q* and *LLR* were still up-regulated by day 14, possibly demonstrating immune recognition occurring in response to ingress of potential pathogens from the surrounding water through the wounds during the slow healing process.

Genes differentially expressed between control and damaged animals were also functionally enriched with processes relating to immune effector mechanisms, for example the GO cellular process melanosome 3 days after damage (FDR = 0.042). In addition, genes up-regulated in damaged animals include those encoding theromacin, a thioster-containing protein and some apoptosis-related proteins. Melanosomes are ‘lysosome-related’ organelles in which melanins are synthesised and stored. It is well known that melanins are a crucial component of the invertebrate immune response (Grimaldi et al. 2012), and parallels have been made between melanosomes in mammalian melanocycte and melanin-rich granules in invertebrate immune cells. Mydlarz et al. (2008), for example, describe melanosomes detected in amoebocytes adjacent to protective melanin bands in infected sea fans, which were absent in uninfected controls. Of particular relevance to the present study, melanin production has repeatedly been demonstrated as an effector mechanism in bivalve immunity (Luna-Acosta et al. 2017). Theromacin is an antimicrobial protein, originally identified in a leech, which is active against Gram-positive bacteria (Tasiemski et al. 2004). A similar gene has been reported for bivalves, which presumably has the same function (Xu et al. 2010). Thioester-containing proteins are a large and diverse group that are present in both vertebrates and invertebrates. In terms of immunity, they are best known as complement factors, C3 and α_2_-macroglobulin. In higher chordates, C3 acts as an opsonin that is deposited on pathogen surfaces, while α_2_-macroglobulin acts as a caging and anti-proteinase molecule (Williams and Baxter 2014). It is likely that the *thioester*-*containing* gene in *M*. *truncata* encodes a protein with similar effects. The up-regulated *apoptosis*-*related* genes are likely to encode proteins involved in programmed cell death, and would be expected to be up-regulated during recovery from drilling, as apoptosis helps to remove redundant and degenerating cells, as well as acting as a defence strategy against viral threats. Taken together, the results discussed above demonstrate that the immune system plays an important role in response to temperature and shell-damage stress in *M. truncata*.

It was surprising that there were no significant differences in the total haemocyte counts between the control and damaged animals (Supplementary Fig. S2, File 3). Previous studies have found that, under stressful conditions (e.g. pollution, acidification and temperature), haemocyte numbers can increase or decrease (Dyrynda et al. 2000; Matozzo et al. 2012). It is possible that any rise in haemogram in the present study may have been masked in the damaged animals by the loss of haemolymph following drilling (through the hole). An alternative explanation could be that the haemocytes migrated to the damage site during the repair process, which has been documented in other bivalves (Mount et al. 2004; Kadar 2008; Li et al. 2016) and hence an active haemocyte production process could have been masked. The exact role of haemocyctes in both normal biomineralisation and shell repair is still unclear; in both scenarios, these cells are likely to be playing a dual role in the immunoprotection of the animal at its external barrier, and in shell production. In addition to total haemocyte numbers, the different haemocyte types (here, DCC) is also an important consideration. Bivalves possess diverse haemocyte types, primarily granular and agranular subpopulations, which perform different immune functions (e.g. Wootton et al. 2003). The DCC under varying conditions, therefore, should ideally be recorded. The low total number of haemocytes in the present study made it impossible to obtain reliable DCC values. Thus, it remains a possibility that there were variations in this parameter, especially as there are always large inter-animal variations in invertebrate animals taken from the wild (Wootton et al. 2003).

Although the primary purpose of the present study was to understand general bivalve cellular stress responses, some of those responses were inevitably shell repair and biomineralisation mechanisms given the specific experimental shell-damage challenge imposed. Inflicting shell damage stimulates calcification pathways to repair the shell and hence, this experimental manipulation has previously been used to study biomineralisation mechanisms (Mount et al. 2004; Fleury et al. 2008; Sleight et al. 2015; Huning et al. 2016). Only three animals produced a brown film with signs of partial calcification to occlude the drill hole (typical of healing category 3) after 14 days. Despite this lack of physiological repair, transcriptomic profiling revealed up-regulated biomineralisation genes. At the 3- and 7-day time points, the protein-protein interaction networks were significantly functionally enriched with cellular processes linked to biomineralisation, such as extracellular regions, membrane-bound vesicles and extracellular exomes (Zhang et al. 2012) (Table 2). In addition, at the 7-day time point, osteoblast differentiation and ossification terms were also functionally enriched, albeit with terminology specific to human biomineralisation as human annotations were used for input into STRING (Table 2). The annotated differentially expressed genes also included putative biomineralisation candidates as previously identified in molluscs (and therefore not in the human-centric STRING database, Supplementary files 3 and 4). Pif is a well-characterised shell matrix protein (Suzuki et al. 2009; Suzuki et al. 2013) that is present in the *M*. *truncata* shell proteome (Arivalagan et al. 2016) and has a mantle-specific expression pattern when compared to other tissues in this species (Sleight et al. 2016). In the present study, *pif* was highlighted as a one of the most significant time-dependant damage-response genes as it was progressively up-regulated in damaged animals throughout the course of the experiment (LogFC values 1 day = 1.4, 3 days = 3.8, 7 days = 4.1 and 14 days = 4.2, Supplementary File 4), and hence, the data presented here provides further evidence for the important role of this gene in molluscan shell biomineralisation. In summary, these analyses show that discovery-led transcriptomic profiling of animals during stress-response experiments can shed light on the complexity of important biological processes and changes within organisms that cannot be detected at higher levels (we could detect biomineralisation signals in the transcriptomic data despite the lack of progress in physiological shell repair).

## Conclusions

Here, we present the first experimental assessment of the cellular response to environmental stressors in an important temperate bivalve species, *M*. *truncata.* Failure to acclimate to increased temperature, as evidenced by the inability to rapidly repair shell damage, suggests that some temperate *M*. *truncata* populations are being pushed past their viable thermal tolerance limit for future ecological sustainability. At the cellular level, this lack of acclimation was clearly associated with key molecular mechanisms, and most specifically the up-regulation of classic stress response genes such as hsps, antioxidants and immune genes.

## Electronic supplementary material

Supplementary Information (PDF 701 kb)

Supplementary File 1 (TXT 7.06 kb)

Supplementary File 2 (CSV 32.9 mb)

Supplementary File 3 (XLSX 87.8 kb)

Supplementary File 4 (XLSX 18.5 kb)

## References

[CR1] Altschul SF, Gish W, Miller W, Myers EW, Lipman DJ (1990). Basic local alignment search tool. J Mol Biol.

[CR2] Amaro T, Duineveld G, Bergman M, Witbaard R (2003). Growth variations in the bivalve Mya truncata: a tool to trace changes in the Frisian front macrofauna (southern North Sea)?. Helgol Mar Res.

[CR3] Amaro T, Duineveld G, Tyler P (2005). Does Mya truncata reproduce at its southern distribution limit? Preliminary information. J Shellfish Res.

[CR4] An J, Shen X, Ma Q, Yang C, Liu S, Chen Y (2014) Transcriptome profiling to discover putative genes associated with Paraquat resistance in Goosegrass (*Eleusine indica* L.). PLoS One 9(6):e99940 10.1371/journal.pone.0099940 PMC4057336 24927422

[CR5] Arivalagan J, Marie B, Sleight VA, Clark MS, Berland S, Marie A (2016). Shell matrix proteins of the clam, Mya truncata: roles beyond shell formation through proteomic study. Mar Genomics.

[CR6] Ballerstedt S (2002) ‘*Mya truncata* blunt gaper’, marine life information network: biology and sensitivity key information reviews [online]. Available: http://www.marlin.ac.uk/species/detail/1626. Accessed 09/12/2016

[CR7] Bay RA, Palumbi SR (2015). Rapid acclimation ability mediated by transcriptome changes in reef-building corals. Genome Biology and Evolution.

[CR8] Bouchereau A, Aziz A, Larher F, Martin-Tanguy J (1999). Polyamines and environmental challenges: recent development. Plant Sci.

[CR9] Caro AA, Cederbaum AI (2004). Oxidative stress, toxicology, and pharmacology of CYP2E1. Annu Rev Pharmacol Toxicol.

[CR10] Chang ES (2005). Stressed-out lobsters: crustacean hyperglycemic hormone and stress proteins. Integr Comp Biol.

[CR11] Checa A (1993). Non-predatory shell damage in recent deep-endobenthic bivalves from Spain. Palaeogeogr Palaeoclimatol Palaeoecol.

[CR12] Clark MS, Peck LS (2009). HSP70 heat shock proteins and environmental stress in Antarctic marine organisms: a mini-review. Mar Genomics.

[CR13] Clark MS, Sommer U, Sihra JK, Thorne MA, Morley SA, King M, Viant MR, Peck LS (2017). Biodiversity in marine invertebrate responses to acute warming revealed by a comparative multi-omics approach. Glob Chang Biol.

[CR14] Cohen AA, de Magalhaes JP, Gohil K (2010). Ecological, biomedical and epidemiological approaches to understanding oxidative balance and ageing: what they can teach each other. Funct Ecol.

[CR15] Csermely P (2004). Strong links are important, but weak links stabilize them. Trends Biochem Sci.

[CR16] Dyrynda EA, Law RJ, Dyrynda PE, Kelly CA, Pipe RK, Ratcliffe NA (2000). Changes in immune parameters of natural mussel Mytilus edulis populations following a major oil spill (‘Sea Empress’, Wales, UK). Mar Ecol Prog Ser.

[CR17] Fink AL (1999). Chaperone-mediated protein folding. Physiol Rev.

[CR18] Fleury C, Marin F, Marie B, Luquet G, Thomas J, Josse C, Serpentini A, Lebel JM (2008). Shell repair process in the green ormer Haliotis tuberculata: a histological and microstructural study. Tissue & Cell.

[CR19] Franceschini A, Szklarczyk D, Frankild S, Kuhn M, Simonovic M, Roth A, Lin JY, Minguez P, Bork P, von Mering C, Jensen LJ (2013). STRING v9.1: protein-protein interaction networks, with increased coverage and integration. Nucleic Acids Res.

[CR20] Frost M, Bayliss-Brown G, Buckley P, Cox M, Dye SR, Sanderson WG, Stoker B, Harvey NW (2016). A review of climate change and the implementation of marine biodiversity legislation in the United Kingdom. Aquatic Conservation-Marine and Freshwater Ecosystems.

[CR21] Gaymer CF, Himmelman JH, Johnson LE (2001). Distribution and feeding ecology of the seastars Leptasterias polaris and Asterias vulgaris in the northern Gulf of St Lawrence, Canada. J Mar Biol Assoc U K.

[CR22] Gaymer CF, Himmelman JH, Johnson LE (2001). Use of prey resources by the seastars Leptasterias polaris and Asterias vulgaris: a comparison between field observations and laboratory experiments. J Exp Mar Biol Ecol.

[CR23] Gerdol M, Venier P, Pallavicini A (2015). The genome of the Pacific oyster Crassostrea gigas brings new insights on the massive expansion of the C1q gene family in Bivalvia. Developmental & Comparative Immunology.

[CR24] Gofas S (2004) ‘World register of marine species(WoRMS): *Mya truncata*’, MolluscaBase (2016) [online]. Available: http://www.marinespecies.org/aphia.php?p=taxdetails&id=140431. Accessed 09/12/2016

[CR25] Grabherr MG, Haas BJ, Yassour M, Levin JZ, Thompson DA, Amit I, Adiconis X, Fan L, Raychowdhury R, Zeng QD, Chen ZH, Mauceli E, Hacohen N, Gnirke A, Rhind N, di Palma F, Birren BW, Nusbaum C, Lindblad-Toh K, Friedman N, Regev A (2011). Full-length transcriptome assembly from RNA-Seq data without a reference genome. Nat Biotechnol.

[CR26] Grimaldi A, Girardello R, Malagoli D, Falabella P, Tettamanti G, Valvassori R, Ottaviani E, de Eguileor M (2012). Amyloid/melanin distinctive mark in invertebrate immunity. Isj-Invertebrate Survival Journal.

[CR27] Haas BJ, Papanicolaou A, Yassour M, Grabherr M, Blood PD, Bowden J, Couger MB, Eccles D, Li B, Lieber M, MacManes MD, Ott M, Orvis J, Pochet N, Strozzi F, Weeks N, Westerman R, William T, Dewey CN, Henschel R, LeDuc RD, Friedman N, Regev A (2013). De novo transcript sequence reconstruction from RNA-Seq: reference generation and analysis with trinity. Nat Protoc.

[CR28] Hanington PC, Zhang SM (2011). The primary role of fibrinogen-related proteins in invertebrates is defense, not coagulation. Journal of Innate Immunity.

[CR29] Hartl FU (1996). Molecular chaperones in cellular protein folding. Nature.

[CR30] Haussmann MF, Marchetto NM (2010). Telomeres: linking stress and survival, ecology and evolution. Current Zoology.

[CR31] Hewitt RA, Dale JE (1984). Growth increments of modern *Mya truncata* L. from the Canadian Arctic, Greenland and Scotland. Geol Surv Can Pap (pt B).

[CR32] Himmelman JH, Dutil C (1991). Distribution, population-structure and feeding of subtidal seastars in the northern golf of St-Lawrence. Mar Ecol Prog Ser.

[CR33] Huang A-M, Geng Y, Wan K-Y, Zeng F, Liu Q, Wang Y, Sun Y, Liu X-X, Zhou Y (2013). Molecular cloning and expression analysis of heat shock protein 90 (Hsp90) of the mud crab, Scylla Paramamosain. J Agric Sci.

[CR34] Huning AK, Lange SM, Ramesh K, Jacob DE, Jackson DJ, Panknin U, Gutowska MA, Philipp EER, Rosenstiel P, Lucassen M, Melzner F (2016). A shell regeneration assay to identify biomineralization candidate genes in mytilid mussels. Mar Genomics.

[CR35] Jones DP, Go YM (2010). Redox compartmentalization and cellular stress. Diabetes Obes Metab.

[CR36] Kádár E (2008). Haemocyte response associated with induction of shell regeneration in the deep-sea vent mussel Bathymodiolus azoricus (Bivalvia: Mytilidae). J Exp Mar Biol Ecol.

[CR37] Korcsmaros T, Kovacs IA, Szalay MS, Csermely P (2007). Molecular chaperones: the modular evolution of cellular networks. J Biosci.

[CR38] Li S, Liu Y, Liu C, Huang J, Zheng G, Xie L, Zhang R (2016). Hemocytes participate in calcium carbonate crystal formation, transportation and shell regeneration in the pearl oyster Pinctada fucata. Fish & shellfish immunology.

[CR39] Lingappan K, Jiang WW, Wang LH, Wang GD, Couroucli XI, Shivanna B, Welty SE, Barrios R, Khan MF, Nebert DW, Roberts LJ, Moorthy B (2014). Mice deficient in the gene for cytochrome P450 (CYP)1A1 are more susceptible than wild-type to Hyperoxic lung injury: evidence for protective role of CYP1A1 against oxidative stress. Toxicol Sci.

[CR40] Luna-Acosta A, Breitwieser M, Renault T, Thomas-Guyon H (2017). Recent findings on phenoloxidases in bivalves. Mar Pollut Bull.

[CR41] Matozzo V, Chinellato A, Munari M, Finos L, Bressan M, Marin MG (2012). First evidence of immunomodulation in bivalves under seawater acidification and increased temperature. PLoS One.

[CR42] McCarthy DJ, Chen YS, Smyth GK (2012). Differential expression analysis of multifactor RNA-Seq experiments with respect to biological variation. Nucleic Acids Res.

[CR43] Morissette S, Himmelman JH (2000). Subtidal food thieves: interactions of four invertebrate kleptoparasites with the sea star Leptasterias polaris. Anim Behav.

[CR44] Mount AS, Wheeler AP, Paradkar RP, Snider D (2004). Hemocyte-mediated shell mineralization in the eastern oyster. Science.

[CR45] Moya A, Huisman L, Foret S, Gattuso JP, Hayward DC, Ball EE, Miller DJ (2015). Rapid acclimation of juvenile corals to CO2-mediated acidification by upregulation of heat shock protein and Bcl-2 genes. Mol Ecol.

[CR46] Mydlarz LD, Holthouse SF, Peters EC, Harvell CD (2008). Cellular responses in sea fan corals: granular Amoebocytes react to pathogen and climate stressors. PLoS One.

[CR47] Noctor G, Foyer CH (1998). Ascorbate and glutathione: keeping active oxygen under control. Annu Rev Plant Physiol Plant Mol Biol.

[CR48] Oliver PG, Holmes AM, Killeen IJ, Turner JA (2016) Marine bivalve shells of the British isles: *Mya truncata* [online]. Available: http://naturalhistory.museumwales.ac.uk/britishbivalves. Accessed 09/12/2016

[CR49] Osovitz CJ, Hofmann GE (2007). Marine macrophysiology: studying physiological variation across large spatial scales in marine systems. Comparative Biochemistry and Physiology a-Molecular & Integrative Physiology.

[CR50] Pancer Z, Amemiya CT, Ehrhardt GR, Ceitlin J, Gartland GL, Cooper MD (2004). Somatic diversification of variable lymphocyte receptors in the agnathan sea lamprey. Nature.

[CR51] Peck LS, Morley SA, Richard J, Clark MS (2014). Acclimation and thermal tolerance in Antarctic marine ectotherms. J Exp Biol.

[CR52] Powell SR, Wang P, Divald A, Teichberg S, Haridas V, McCloskey TW, Davies KJA, Katzeff H (2005). Aggregates of oxidized proteins (lipofuscin) induce apoptosis through proteasome inhibition and dysregulation of proapoptotic proteins. Free Radic Biol Med.

[CR53] Queiros AM, Birchenough SNR, Bremner J, Godbold JA, Parker RE, Romero-Ramirez A, Reiss H, Solan M, Somerfield PJ, Van Colen C, Van Hoey G, Widdicombe S (2013). A bioturbation classification of European marine infaunal invertebrates. Ecology and Evolution.

[CR54] Robinson MD, McCarthy DJ, Smyth GK (2010). edgeR: a Bioconductor package for differential expression analysis of digital gene expression data. Bioinformatics.

[CR55] Sagarin RD, Somero GN (2006). Complex patterns of expression of heat-shock protein 70 across the southern biogeographical ranges of the intertidal mussel Mytilus californianus and snail Nucella ostrina. J Biogeogr.

[CR56] Schill RO, Gorlitz H, Kohler HR (2003). Laboratory simulation of a mining accident: acute toxicity, hsc/hsp70 response, and recovery from stress in Gammarus fossarum (Crustacea, Amphipoda) exposed to a pulse of cadmium. Biometals.

[CR57] Sleight VA, Thorne MA, Peck LS, Clark MS (2015). Transcriptomic response to shell damage in the Antarctic clam, Laternula elliptica: time scales and spatial localisation. Mar Genomics.

[CR58] Sleight VA, Thorne MAS, Peck LS, Arivalagan J, Berland S, Marie A, Clark MS (2016). Characterisation of the mantle transcriptome and biomineralisation genes in the blunt-gaper clam, Mya truncata. Mar Genomics.

[CR59] Somero GN (2010). The physiology of climate change: how potentials for acclimatization and genetic adaptation will determine ‘winners' and ‘losers’. J Exp Biol.

[CR60] Somero GN (2012). The physiology of global change: linking patterns to mechanisms. Annu Rev Mar Sci.

[CR61] Suzuki M, Saruwatari K, Kogure T, Yamamoto Y, Nishimura T, Kato T, Nagasawa H (2009). An acidic matrix protein, Pif, is a key macromolecule for nacre formation. Science.

[CR62] Suzuki M, Iwashima A, Kimura M, Kogure T, Nagasawa H (2013). The molecular evolution of the pif family proteins in various species of mollusks. Mar Biotechnol.

[CR63] Szklarczyk D, Franceschini A, Wyder S, Forslund K, Heller D, Huerta-Cepas J, Simonovic M, Roth A, Santos A, Tsafou KP, Kuhn M, Bork P, Jensen LJ, von Mering C (2015). STRING v10: protein-protein interaction networks, integrated over the tree of life. Nucleic Acids Res.

[CR64] Tasiemski A, Vandenbulcke F, Mitta G, Lemoine J, Lefebvre C, Sautiere PE, Salzet M (2004). Molecular characterization of two novel antibacterial peptides inducible upon bacterial challenge in an annelid, the leech Theromyzon tessulatum. J Biol Chem.

[CR65] Wang L, Wang L, Zhang H, Zhou Z, Siva VS, Song L (2012). A C1q domain containing protein from scallop Chlamys farreri serving as pattern recognition receptor with heat-aggregated IgG binding activity. PLoS One.

[CR66] Welch HE, Bergmann MA, Siferd TD, Martin KA, Curtis MF, Crawford RE, Conover RJ, Hop H (1992). Energy-flow through the marine ecosystem of the Lancaster Sound region Arctic Canada. Arctic.

[CR67] Williams M, Baxter R (2014). The structure and function of thioester-containing proteins in arthropods. Biophys Rev.

[CR68] Witbaard R, Duineveld GCA, Amaro T, Bergman MJN (2005). Growth trends in three bivalve species indicate climate forcing on the benthic ecosystem in the southeastern North Sea. Clim Res.

[CR69] Wootton EC, Dyrynda EA, Ratcliffe NA (2003). Bivalve immunity: comparisons between the marine mussel (Mytilus edulis), the edible cockle (Cerastoderma edule) and the razor-shell (Ensis siliqua). Fish Shellfish Immunol.

[CR70] Wu GY, Fang YZ, Yang S, Lupton JR, Turner ND (2004). Glutathione metabolism and its implications for health. J Nutr.

[CR71] Xu QQ, Wang GL, Yuan HW, Chai Y, Xiao ZL (2010). cDNA sequence and expression analysis of an antimicrobial peptide, theromacin, in the triangle-shell pearl mussel Hyriopsis cumingii. Comparative Biochemistry Physiology B-Biochemistry Molecular Biology.

[CR72] Yang W, Shen YJ, Chen Y, Chen L, Wang L, Wang HP, Xu SC, Fang SY, Fu Y, Yu YQ, Shen YX (2014). Mesencephalic astrocyte-derived neurotrophic factor prevents neuron loss via inhibiting ischemia-induced apoptosis. J Neurol Sci.

[CR73] Zhang H, Song L, Li C, Zhao J, Wang H, Qiu L, Ni D, Zhang Y (2008). A novel C1q-domain-containing protein from Zhikong scallop Chlamys farreri with lipopolysaccharide binding activity. Fish Shellfish Immunol.

[CR74] Zhang G, Fang X, Guo X, Li L, Luo R, Xu F, Yang P, Zhang L, Wang X, Qi H, Xiong Z, Que H, Xie Y, Holland PWH, Paps J, Zhu Y, Wu F, Chen Y, Wang J, Peng C, Meng J, Yang L, Liu J, Wen B, Zhang N, Huang Z, Zhu Q, Feng Y, Mount A, Hedgecock D, Xu Z, Liu Y, Domazet-Loso T, Du Y, Sun X, Zhang S, Liu B, Cheng P, Jiang X, Li J, Fan D, Wang W, Fu W, Wang T, Wang B, Zhang J, Peng Z, Li Y, Li N, Wang J, Chen M, He Y, Tan F, Song X, Zheng Q, Huang R, Yang H, Du X, Chen L, Yang M, Gaffney PM, Wang S, Luo L, She Z, Ming Y, Huang W, Zhang S, Huang B, Zhang Y, Qu T, Ni P, Miao G, Wang J, Wang Q, Steinberg CEW, Wang H, Li N, Qian L, Zhang G, Li Y, Yang H, Liu X, Wang J, Yin Y, Wang J (2012). The oyster genome reveals stress adaptation and complexity of shell formation. Nature.

[CR75] Zheng L, Zhang L, Lin H, McIntosh M, Malacrida A (2005). Toll-like receptors in invertebrate innate immunity. Invertebrate Survival J.

[CR76] Ziuganov V, San Miguel E, Neves RJ, Longa A, Fernandez C, Amaro R, Beletsky V, Popkovitch E, Kaliuzhin S, Johnson T (2000). Life span variation of the freshwater pearl shell: a model species for testing longevity mechanisms in animals. Ambio.

