# Supplementary material for: Cellular stress responses to chronic heat shock and shell damage in temperate Mya truncata
Source: Cell Stress Chaperones. 2018 May 12;23(5):1003–17. doi: 10.1007/s12192-018-0910-5 (PMC6111077; doi:10.1007/s12192-018-0910-5)
Supplement: Supplementary file 1 — (PDF 701 kb) [file 12192_2018_910_MOESM1_ESM.pdf]

Cellular stress responses to chronic heat shock and shell damage in temperate *Mya truncata*

Victoria A. Sleight[1,2]*, Lloyd S. Peck[2], Elisabeth A. Dyrynda[3], Valerie J. Smith[4] and Melody S. Clark[2]

[1]Department of Zoology, University of Cambridge, Downing Street, Cambridge, CB2 3EJ, UK

[2]British Antarctic Survey, Natural Environment Research Council (NERC), High Cross, Madingley Road, Cambridge, CB3 0ET, UK.

[3]Centre for Marine Biodiversity & Biotechnology, Institute of Life & Earth Sciences, Heriot-Watt University, Edinburgh, EH14 4AS, UK

[4]Scottish Oceans Institute, School of Biology, University of St Andrews, St Andrews, Fife, KY16 8LB, UK

*Corresponding author: Victoria A. Sleight, [1]Department of Zoology, University of Cambridge, Downing Street, Cambridge, CB2 3EJ, UK. email: vas45@cam.ac.uk

<u>Supplementary Information:</u>

Materials and Methods

Trinity quality checking

As per the main manuscript, transcript abundance estimation was quality checked using the Trinity Perl-to-R ‘PtR’ toolkit, specifically principle component analysis was used to check for batch effect and outliers [Figure S1. (Haas *et al.*, 2013)]. Three clear outliers were identified (Figure S1a) and upon further inspection of the libraries (which showed they were orders of magnitude smaller than the other libraries) they were removes. Once outlying libraries were removed from the PCA analysis, clear patterns in time*treatment (Figure S1b), time (Figure S1c) and treatment (Supplementary Figure S1d). Importantly, individual libraries clustered with their respective time*treatment category (Figure S1b-d) and there was no indication of batch effects.

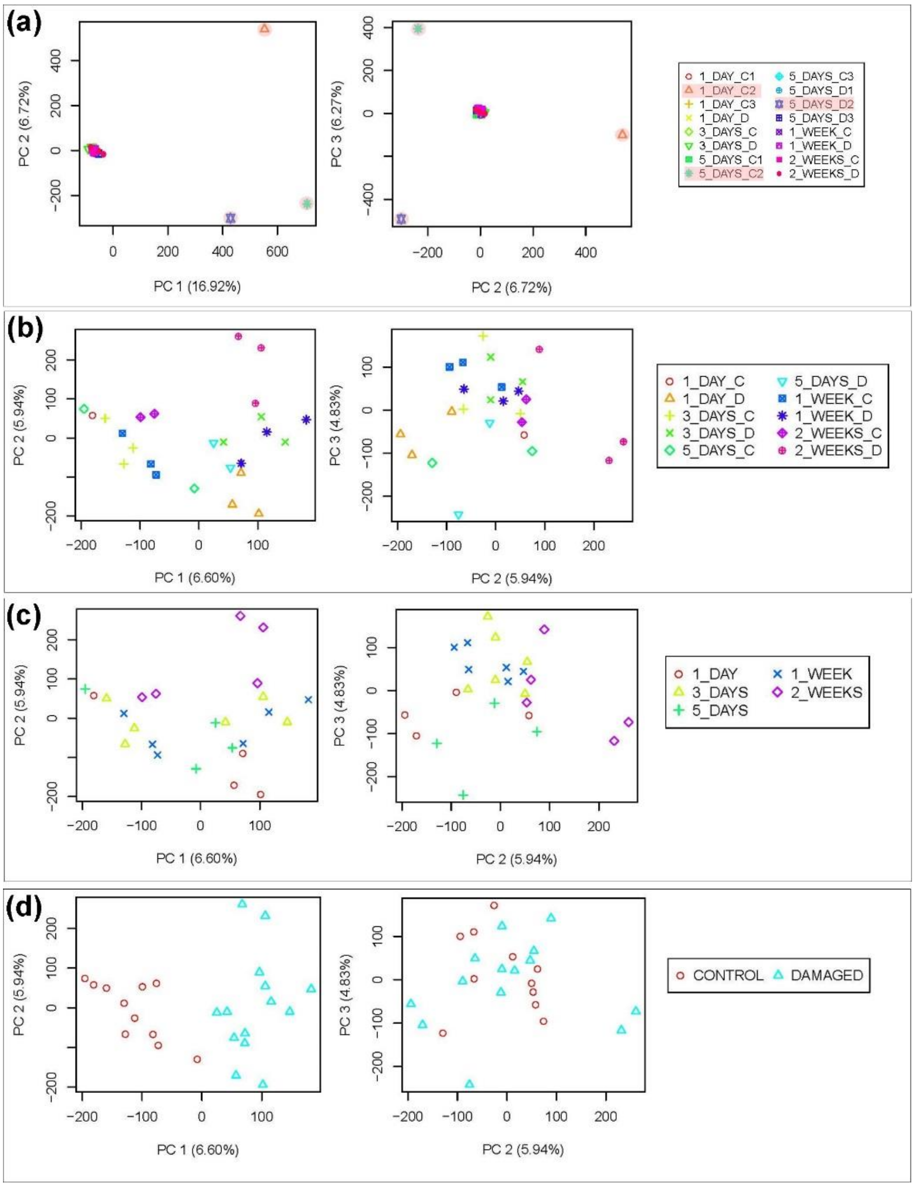

Supplementary Figure S1. Principle component analysis of transcript abundance estimation for each library. **(a)** All libraries included and 3 outliers identified (highlighted in red). **(b)** Three outliers removed and labelled as per treatment*time. **(c)** Three outliers removed and labelled as per treatment. **(d)** Three outliers removed and labelled as per treatment.

Results:

There was no difference in total haemocyte cell counts between control and damaged individuals at any time point (Figure S3).

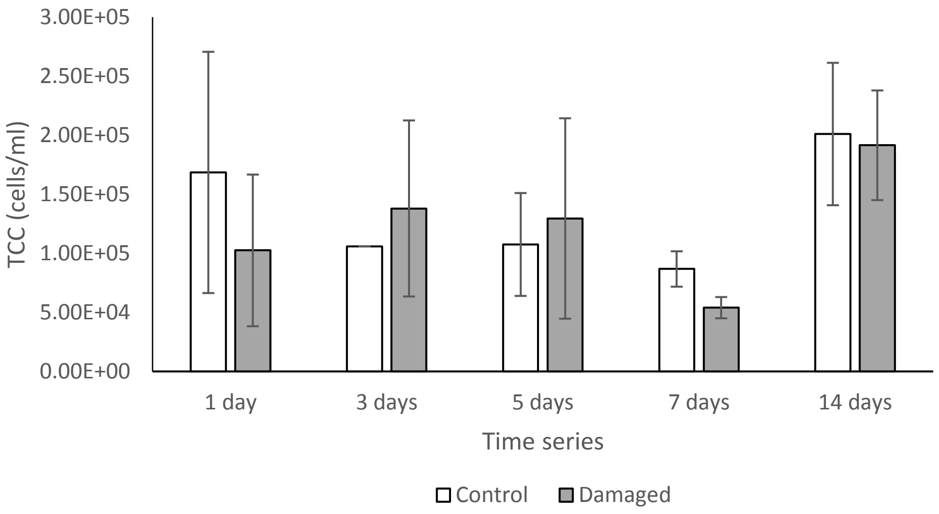

Supplementary Figure S2. Total circulating haemocyte cells per ml of haemolymph (mean ±SE, n = 4) in damaged and control animals at each time point.

As per methods section of the main manuscript, to aid interpretation of the hundreds of up and down regulated genes, and to provide visual qualitative assessment of the differential biological processes at each time point, STRING (v10.0) was used to produce protein-protein interaction networks. All of the differently expressed genes (FDR<0.05) at each time point were compared to a local UniProt/SwissProt human database (updated 05 October 2016) using Basic Local Alignment Search Tool (blastx, cut-off $<1e^{-10}$). Following human annotation gene IDs were then inputted into STRING to build protein-protein interaction networks. The IDs for the human annotations can be found in table S1.

Supplementary Table S1. Abbreviations used in STRING protein-protein interaction networks (Figure 6, main manuscript).

**Abbreviation**	**Full Uniprot/Swissprot name**
DDX1	DEAD (Asp-Glu-Ala-Asp) box helicase 1
ABCF2	ATP-binding cassette, sub-family F (GCN20), member 2 (634 aa)
ABCF2	ATP-binding cassette, sub-family F (GCN20), member 2 (634 aa)
ABCF2	ATP-binding cassette, sub-family F (GCN20), member 2 (634 aa)
ABCF3	ATP-binding cassette, sub-family F (GCN20), member 3

ABHD15	abhydrolase domain containing 15 (468 aa)
ACSF2	acyl-CoA synthetase family member 2
ACSL5	acyl-CoA synthetase long-chain family member 5
ACTG1	actin, gamma 1
ADAMTS9	ADAM metallopeptidase with thrombospondin type 1 motif, 9
ADAR	adenosine deaminase, RNA-specific (1226 aa)
AGL	amylo-alpha-1, 6-glucosidase, 4-alpha-glucanotransferase (1532 aa)
ALDH1B1	aldehyde dehydrogenase 1 family, member B1
ALG8	asparagine-linked glycosylation 8, alpha-1,3-glucosyltransferase homolog
ALKBH2	alkB, alkylation repair homolog 2
ALKBH6	alkB, alkylation repair homolog 6
AMBP	alpha-1-microglobulin/bikunin precursor
AMY2A	amylase, alpha 2A (pancreatic) (511 aa)
AMY2B	amylase, alpha 2B (pancreatic) (511 aa)
ANAPC4	anaphase promoting complex subunit 4
ANGPTL6	angiopoietin-like 6
ANK2	ankyrin 2, neuronal
APEH	N-acylaminoacyl-peptide hydrolase
AQP4	aquaporin 4
ARHGEF10	Rho guanine nucleotide exchange factor (GEF) 10
ARIH1	ariadne homolog, ubiquitin-conjugating enzyme E2 binding protein, 1
ASNS	asparagine synthetase (glutamine-hydrolyzing) (561 aa)
ASRGL1	asparaginase like 1
ATAD3A	ATPase family, AAA domain containing 3A
ATP1A1	ATPase, Na+/K+ transporting, alpha 1 polypeptide
ATPAF2	ATP synthase mitochondrial F1 complex assembly factor 2
BCL2L1	BCL2-like 1
BIRC2	baculoviral IAP repeat containing 2
BIRC8	baculoviral IAP repeat containing 8
BOD1	biorientation of chromosomes in cell division 1
BRD8	bromodomain containing 8
BVES	blood vessel epicardial substance
C1QL3	complement component 1, q subcomponent-like 3
C8orf82	chromosome 8 open reading frame 82 (216 aa)
CABIN1	calcineurin binding protein 1
CALM2	calmodulin 2 (phosphorylase kinase, delta) (149 aa)
CALR	calreticulin
CALU	calumenin (323 aa)
CARS	cysteinyl-tRNA synthetase (831 aa)
CASP7	caspase 7, apoptosis-related cysteine peptidase (336 aa)
CASP9	caspase 9, apoptosis-related cysteine peptidase (416 aa)
CBFB	core-binding factor, beta subunit
CCNB3	cyclin B3
CD109	CD109 molecule (1445 aa)

CD151	CD151 molecule (Raph blood group)
CD163L1	CD163 molecule-like 1 (1453 aa)
CDA	cytidine deaminase
CDC42	cell division cycle 42 (GTP binding protein, 25kDa)
CEBPG	CCAAT/enhancer binding protein (C/EBP), gamma
CEBPZ	CCAAT/enhancer binding protein (C/EBP), zeta
CERK	ceramide kinase
CH25H	cholesterol 25-hydroxylase
CHAC1	ChaC, cation transport regulator homolog 1 (E. coli)
CHDH	choline dehydrogenase (594 aa)
CHRNA10	cholinergic receptor, nicotinic, alpha 10 (neuronal)
CHRNA2	cholinergic receptor, nicotinic, alpha 2 (neuronal)
CHRNA6	cholinergic receptor, nicotinic, alpha 6 (neuronal)
CHRNB3	cholinergic receptor, nicotinic, beta 3 (neuronal)
CHRNB3	cholinergic receptor, nicotinic, beta 3 (neuronal)
CHST8	carbohydrate (N-acetylgalactosamine 4-0) sulfotransferase 8
CILP	cartilage intermediate layer protein, nucleotide pyrophosphohydrolase
CLCN6	chloride channel, voltage-sensitive 6 (869 aa)
CLEC7A	C-type lectin domain family 7, member A (247 aa)
COL6A3	collagen, type VI, alpha 3
COL6A5	collagen, type VI, alpha 5 (2615 aa)
CPAMD8	C3 and PZP-like, alpha-2-macroglobulin domain containing 8 (1932 aa)
CPNE1	copine I
CREB1	cAMP responsive element binding protein 1
CRYAB	crystallin, alpha B
CTBS	chitobiase, di-N-acetyl-
CTRL	chymotrypsin-like (264 aa)
CTSC	cathepsin C (463 aa)
CTSK	cathepsin K
CYHR1	cysteine/histidine-rich 1 (362 aa)
CYP1B1	cytochrome P450, family 1, subfamily B, polypeptide 1
CYP20A1	cytochrome P450, family 20, subfamily A, polypeptide 1 (462 aa)
CYP2C18	cytochrome P450, family 2, subfamily C, polypeptide 18
CYP2J2	cytochrome P450, family 2, subfamily J, polypeptide 2
CYP2R1	cytochrome P450, family 2, subfamily R, polypeptide 1
CYP2U1	cytochrome P450, family 2, subfamily U, polypeptide 1
CYP3A4	cytochrome P450, family 3, subfamily A, polypeptide 4
DBH	dopamine beta-hydroxylase (dopamine beta-monooxygenase)
DBI	diazepam binding inhibitor (GABA receptor modulator, acyl-CoA binding protein) (148 aa)
DDR2	discoidin domain receptor tyrosine kinase 2
DDX31	DEAD (Asp-Glu-Ala-Asp) box polypeptide 31 (851 aa)
DHX8	DEAH (Asp-Glu-Ala-His) box polypeptide 8
DIS3L2	DIS3 mitotic control homolog -like 2
DMRTA2	DMRT-like family A2

DNAJB11	DnaJ (Hsp40) homolog, subfamily B, member 11
DOT1L	DOT1-like, histone H3 methyltransferase
DPY19L3	dpy-19-like 3
DTX3L	deltex 3-like
DUSP10	dual specificity phosphatase 10
DUSP4	dual specificity phosphatase 4
DYNC1H1	dynein, cytoplasmic 1, heavy chain 1
ECHDC3	enoyl CoA hydratase domain containing 3 (303 aa)
EEF1G	eukaryotic translation elongation factor 1 gamma
EGR3	early growth response 3
EIF2B2	eukaryotic translation initiation factor 2B, subunit 2 beta, 39kDa
EIF2S2	eukaryotic translation initiation factor 2, subunit 2 beta, 38kDa
EIF3L	eukaryotic translation initiation factor 3, subunit L
EIF4A1	eukaryotic translation initiation factor 4A1
EIF4E	eukaryotic translation initiation factor 4E
EIF5	eukaryotic translation initiation factor 5
ELAVL4	ELAV (embryonic lethal, abnormal vision-like 4
EN1	engrailed homeobox 1 (392 aa)
ENDOU	endonuclease, polyU-specific
ENTPD3	ectonucleoside triphosphate diphosphohydrolase 3
EPRS	glutamyl-prolyl-tRNA synthetase (1512 aa)
ERI3	ERI1 exoribonuclease family member 3 (337 aa)
ESR1	estrogen receptor 1
EVA1C	eva-1 homolog C
FADS2	fatty acid desaturase 2
FAM204A	family with sequence similarity 204, member A (233 aa)
FAM221A	family with sequence similarity 221, member A (298 aa)
FAM63A	family with sequence similarity 63, member A (517 aa)
FAM69C	family with sequence similarity 69, member C (419 aa)
FASN	fatty acid synthase (2511 aa)
FBL	fibrillarin
FBN1	fibrillin 1
FBXO32	F-box protein 32
FBXO33	F-box protein 33
FBXW4	F-box and WD repeat domain containing 4
FBXW7	F-box and WD repeat domain containing 7, E3 ubiquitin protein ligase
FCGBP	Fc fragment of IgG binding protein
FCHSD2	FCH and double SH3 domains 2 (740 aa)
FCN3	ficolin (collagen/fibrinogen domain containing) 3 (Hakata antigen)
FGFR1	fibroblast growth factor receptor 1 (853 aa)
FGFRL1	fibroblast growth factor receptor-like 1
FIBCD1	fibrinogen C domain containing 1
FICD	FIC domain containing
FLVCR2	feline leukemia virus subgroup C cellular receptor family, member 2

FMO6P	flavin containing monooxygenase 6 pseudogene
FOXP2	forkhead box P2 (740 aa)
FRK	fyn-related kinase
GADD45B	growth arrest and DNA-damage-inducible, beta
GAL3ST1	galactose-3-O-sulfotransferase 1
GAL3ST3	galactose-3-O-sulfotransferase 3
GALM	galactose mutarotase (aldose 1-epimerase)
GANAB	glucosidase, alpha
GARNL3	GTPase activating Rap/RanGAP domain-like 3 (1013 aa)
GFM1	G elongation factor, mitochondrial 1
GIN1	gypsy retrotransposon integrase 1 (522 aa)
GLRA3	glycine receptor, alpha 3
GLT25D1	glycosyltransferase 25 domain containing 1
GMPPB	GDP-mannose pyrophosphorylase B (387 aa)
GNPNAT1	glucosamine-phosphate N-acetyltransferase 1 (184 aa)
GNPTAB	N-acetylglucosamine-1-phosphate transferase, alpha and beta subunits
GP5	glycoprotein V (platelet)
GPSM2	G-protein signaling modulator 2
GRM7	glutamate receptor, metabotropic 7
GRPEL1	GrpE-like 1, mitochondrial
GSN	gelsolin
GSS	glutathione synthetase (474 aa)
GTPBP2	GTP binding protein 2 (602 aa)
HECA	headcase homolog (Drosophila)
HEXA	hexosaminidase A (alpha polypeptide)
HGSNAT	heparan-alpha-glucosaminide N-acetyltransferase
HM13	histocompatibility (minor) 13 (426 aa)
HMCN1	hemicentin 1 (5635 aa)
HMGCL	3-hydroxymethyl-3-methylglutaryl-CoA lyase
HPD	4-hydroxyphenylpyruvate dioxygenase
HSD17B14	hydroxysteroid (17-beta) dehydrogenase 14
HSPA12A	heat shock 70kDa protein 12A (675 aa)
HSPA5	heat shock 70kDa protein 5 (glucose-regulated protein, 78kDa)
HSPA8	heat shock 70kDa protein 8
HSPB2	HSPB2-C11orf52 readthrough (non-protein coding)
HSPD1	heat shock 60kDa protein 1 (chaperonin)
HSPE1	heat shock 10kDa protein 1 (chaperonin 10)
HYOU1	hypoxia up-regulated 1
IARS	isoleucyl-tRNA synthetase (1262 aa)
IFI44L	interferon-induced protein 44-like
IGFALS	insulin-like growth factor binding protein, acid labile subunit
IGSF10	immunoglobulin superfamily, member 10
IMPDH1	IMP (inosine 5?-monophosphate) dehydrogenase 1
IMPDH2	IMP (inosine 5?-monophosphate) dehydrogenase 2

IRGC	immunity-related GTPase family, cinema (463 aa)
ISYNA1	inositol-3-phosphate synthase 1 (558 aa)
JMJD6	jumonji domain containing 6
JUND	jun D proto-oncogene
KAT5	K(lysine) acetyltransferase 5 (546 aa)
KCNA1	potassium voltage-gated channel, shaker-related subfamily, member 1 (episodic ataxia with myokymia)
KCTD7	potassium channel tetramerisation domain containing 7
KIAA1161	KIAA1161
KIAA1456	KIAA1456 (454 aa)
KLF7	Kruppel-like factor 7 (ubiquitous)
KLHL29	kelch-like 29 (875 aa)
KYNU	kynureninase
LHX1	LIM homeobox 1
LIMA1	LIM domain and actin binding 1 (760 aa)
LIPA	lipase A, lysosomal acid, cholesterol esterase
LMO4	LIM domain only 4
LNPEP	leucyl/cystinyl aminopeptidase
LONP2	lon peptidase 2, peroxisomal
LOXL3	lysyl oxidase-like 3 (753 aa)
LPIN1	lipin 1
LRIG1	leucine-rich repeats and immunoglobulin-like domains 1
LRIG3	leucine-rich repeats and immunoglobulin-like domains 3
LRP4	low density lipoprotein receptor-related protein 4
MAEA	macrophage erythroblast attacher
MANF	mesencephalic astrocyte-derived neurotrophic factor
MAP2K3	mitogen-activated protein kinase kinase 3
MATN1	matrilin 1, cartilage matrix protein
MDGA1	MAM domain containing glycosylphosphatidylinositol anchor 1
MEIS2	Meis homeobox 2 (477 aa)
MEIS2	Meis homeobox 2 (477 aa)
MFAP4	microfibrillar-associated protein 4
MFAP4	microfibrillar-associated protein 4
MMAB	methylmalonic aciduria (cobalamin deficiency) cblB type (250 aa)
MME	membrane metallo-endopeptidase
MME	membrane metallo-endopeptidase
MMP25	matrix metallopeptidase 25
MOXD1	monooxygenase, DBH-like 1 (613 aa)
MRC1	mannose receptor, C type 1
MRC2	mannose receptor, C type 2
MRPL9	mitochondrial ribosomal protein L9 (267 aa)
MRPS17	mitochondrial ribosomal protein S17 (130 aa)
MSH6	mutS homolog 6 (E. coli)
MSRA	methionine sulfoxide reductase A
MUT	methylmalonyl CoA mutase

MYH4	myosin, heavy chain 4, skeletal muscle
N6AMT2	N-6 adenine-specific DNA methyltransferase 2 (putative)
NDUFAF3	NADH dehydrogenase (ubiquinone) complex I, assembly factor 3
NEGR1	neuronal growth regulator 1
NETO1	neuropilin (NRP) and tolloid (TLL)-like 1
NFIL3	nuclear factor, interleukin 3 regulated
NFKBIA	nuclear factor of kappa light polypeptide gene enhancer in B-cells inhibitor, alpha
NFKBID	nuclear factor of kappa light polypeptide gene enhancer in B-cells inhibitor, delta
NHP2L1	NHP2 non-histone chromosome protein 2-like 1 (S. cerevisiae)
NOA1	nitric oxide associated 1
NOG	noggin
NOTCH1	notch 1
NPC1	Niemann-Pick disease, type C1
NPEPPS	aminopeptidase puromycin sensitive
NR1D2	nuclear receptor subfamily 1, group D, member 2
NUP98	nucleoporin 98kDa (1800 aa)
NXPE3	neurexophilin and PC-esterase domain family, member 3 (559 aa)
OGFOD1	2-oxoglutarate and iron-dependent oxygenase domain containing 1 (542 aa)
P4HA1	prolyl 4-hydroxylase, alpha polypeptide I
P4HA2	prolyl 4-hydroxylase, alpha polypeptide II
P4HA3	prolyl 4-hydroxylase, alpha polypeptide III
PAH	phenylalanine hydroxylase (452 aa)
PAM	peptidylglycine alpha-amidating monooxygenase (974 aa)
PAOX	polyamine oxidase (exo-N4-amino) (511 aa)
PARP15	poly (ADP-ribose) polymerase family, member 15
PCBP3	poly(rC) binding protein 3
PCDH11X	protocadherin 11 X-linked (1347 aa)
PDE10A	phosphodiesterase 10A
PDIA3	protein disulfide isomerase family A, member 3 (505 aa)
PDIA6	protein disulfide isomerase family A, member 6
PIBF1	progesterone immunomodulatory binding factor 1
PIM1	pim-1 oncogene
PNLIP	pancreatic lipase (465 aa)
PNLIPRP1	pancreatic lipase-related protein 1
POLR2E	polymerase (RNA) II (DNA directed) polypeptide E, 25kDa
PPFIBP1	PTPRF interacting protein, binding protein 1 (liprin beta 1)
PPIA	peptidylprolyl isomerase A (cyclophilin A)
PRDX6	peroxiredoxin 6
PRSS12	protease, serine, 12 (neurotrypsin, motopsin)
PTPRT	protein tyrosine phosphatase, receptor type, T (1441 aa)
PXDN	peroxidasin homolog
RASEF	RAS and EF-hand domain containing
RDBP	RD RNA binding protein
RHOBTB1	Rho-related BTB domain containing 1 (696 aa)

RHOBTB3	Rho-related BTB domain containing 3
RND3	Rho family GTPase 3
RNF121	ring finger protein 121 (327 aa)
RNF170	ring finger protein 170 (258 aa)
ROBO1	roundabout, axon guidance receptor, homolog 1
RPL19	ribosomal protein L19 (196 aa)
RPS26	ribosomal protein S26 (115 aa)
RPS27L	ribosomal protein S27-like (84 aa)
RPUSD1	RNA pseudouridylate synthase domain containing 1 (312 aa)
RTN4RL1	reticulon 4 receptor-like 1
RUNX1	runt-related transcription factor 1 (480 aa)
SAA2	serum amyloid A2
SCRT2	scratch homolog 2, zinc finger protein
SDF2	stromal cell-derived factor 2 (211 aa)
SELENBP1	selenium binding protein 1
SELP	selectin P (granule membrane protein 140kDa, antigen CD62)
SESN3	sestrin 3 (492 aa)
SF3B3	splicing factor 3b, subunit 3, 130kDa
SH3GL3	SH3-domain GRB2-like 3
SIAH1	siah E3 ubiquitin protein ligase 1
SLC13A3	solute carrier family 13 (sodium-dependent dicarboxylate transporter), member 3 (602 aa)
SLC16A14	solute carrier family 16, member 14 (monocarboxylic acid transporter 14)
SLC16A7	solute carrier family 16, member 7 (monocarboxylic acid transporter 2)
SLC17A5	solute carrier family 17 (anion/sugar transporter), member 5
SLC22A3	solute carrier family 22 (extraneuronal monoamine transporter), member 3
SLC34A2	solute carrier family 34 (sodium phosphate), member 2
SLC46A3	solute carrier family 46, member 3 (463 aa)
SLC4A10	solute carrier family 4, sodium bicarbonate transporter, member 10
SLC5A6	solute carrier family 5 (sodium-dependent vitamin transporter), member 6
SLC5A8	solute carrier family 5 (iodide transporter), member 8
SLC6A19	solute carrier family 6 (neutral amino acid transporter), member 19
SLC7A8	solute carrier family 7 (amino acid transporter light chain, L system), member 8
SLC8A3	solute carrier family 8 (sodium/calcium exchanger), member 3
SLCO4A1	solute carrier organic anion transporter family, member 4A1
SLIT1	slit homolog 1
SLIT3	slit homolog 3
SNRPB2	small nuclear ribonucleoprotein polypeptide B
SOCS2	suppressor of cytokine signaling 2
SOD1	superoxide dismutase 1, soluble
SOD1	superoxide dismutase 1, soluble
SORD	sorbitol dehydrogenase
SPDEF	SAM pointed domain containing ets transcription factor
SPDEF	SAM pointed domain containing ets transcription factor
SPON2	spondin 2, extracellular matrix protein

SRSF1	serine/arginine-rich splicing factor 1
SRSF1	serine/arginine-rich splicing factor 1
SRSF4	serine/arginine-rich splicing factor 4
STOML2	stomatin (EPB72)-like 2
STT3B	STT3, subunit of the oligosaccharyltransferase complex, homolog B
STXBP4	syntaxin binding protein 4
SUCLG2	succinate-CoA ligase, GDP-forming, beta subunit
SULT1A1	sulfotransferase family, cytosolic, 1A, phenol-preferring, member 1
SVEP1	sushi, von Willebrand factor type A, EGF and pentraxin domain containing 1 (3571 aa)
TAF1A	TATA box binding protein (TBP)-associated factor, RNA polymerase I, A, 48kDa
TAL1	T-cell acute lymphocytic leukemia 1
TAT	tyrosine aminotransferase
TBK1	TANK-binding kinase 1
TFIP11	tuftelin interacting protein 11
TFPI	tissue factor pathway inhibitor (lipoprotein-associated coagulation inhibitor)
TLL2	tolloid-like 2
TLR1	toll-like receptor 1
TMED7	transmembrane emp24 protein transport domain containing 7
TMED9	transmembrane emp24 protein transport domain containing 9
TNC	tenascin C
TNR	tenascin R
TPO	thyroid peroxidase (933 aa)
TRAF4	TNF receptor-associated factor 4
TRHDE	thyrotropin-releasing hormone degrading enzyme
TRMT112	tRNA methyltransferase 11-2 homolog
TSPAN7	tetraspanin 7
TSSK2	testis-specific serine kinase 2
TTC36	tetratricopeptide repeat domain 36 (189 aa)
TYMP	thymidine phosphorylase
UBA1	ubiquitin-like modifier activating enzyme 1
UBA5	ubiquitin-like modifier activating enzyme 5
UBA52	ubiquitin A-52 residue ribosomal protein fusion product 1 (128 aa)
UBB	ubiquitin B (229 aa)
UBE2M	ubiquitin-conjugating enzyme E2M
UGGT1	UDP-glucose glycoprotein glucosyltransferase 1
UNC79	unc-79 homolog
UNG	uracil-DNA glycosylase
USH1C	Usher syndrome 1C (autosomal recessive, severe)
VARS	valyl-tRNA synthetase (1264 aa)
VAT1L	vesicle amine transport protein 1 homolog-like (419 aa)
VRTN	vertebrae development homolog (702 aa)
WARS	tryptophanyl-tRNA synthetase
XDH	xanthine dehydrogenase
XPO1	exportin 1 CRM1 homolog

XRCC6	X-ray repair complementing defective repair
YIF1B	Yip1 interacting factor homolog B (314 aa)
YRDC	yrdC domain containing
ZC4H2	zinc finger, C4H2 domain containing (224 aa)
ZNF121	zinc finger protein 121
ZNF362	zinc finger protein 362
ZNF479	zinc finger protein 479
ZNF57	zinc finger protein 57
ZNF706	zinc finger protein 706 (76 aa)
ZSCAN12	zinc finger and SCAN domain containing 12
ZSWIM6	zinc finger, SWIM-type containing 6 (1215 aa)

Discussion

```
                                                Motif I

TRINITY_DN156808_c2_g1_1      MSASG--PAIGIDLGTTYSCVGVFQHGKVEIIANDQGNRTTPSYVAFTDS
Mya_contig00268               MADKGKAPAVGIDLGTTYSCVGVFQHGKVEIIANDQGNRTTPSYVAFTDT
                              *: .*  **:***************************************:
TRINITY_DN156808_c2_g1_1      ERLLGDAAKNQAAMNPQNTVFDAKRLIGRDFSESNVQSDIKHWPFKVINA
Mya_contig00268               ERLIGDAAKNQVAMNPSNTIFDAKRLIGRKFDDANVQADMKHWPFEVAND
                              ***:*******.****.**:*********.*.::***:*:*****:* *
                                                                     SBD
TRINITY_DN156808_c2_g1_1      GGKPKIQTEYKGETKTFAPEEISSMVLTKMRETAEAYLGKNVTNAVVTVP
Mya_contig00268               AGKPKLKVDYKGDQKTFFPEEVSSMVLLKMKETAEAYLGKTVTNAVITVP
                              .****::.:***: *** ***:***** **:*********.*****:***
Motif III                 Motif IV
TRINITY_DN156808_c2_g1_1      AYFNDAQRRATKDAGAIAGLNVMRIINEPTAAALAYGLDKNLSGEKNVLI
Mya_contig00268               AYFNDSQRQATKDAGTISGMNILRIINEPTAAAIAYGLDKKVGGERNVLI
                              *****:**:******:*:*:*::**********:******::.**:****
  Motif II
TRINITY_DN156808_c2_g1_1      FDLGGGTFDVSILTIDEGSLFEVRSTAGDTHLGGEDFDNRMVNHFIQEFK
Mya_contig00268               FDLGGGTFDVSVLTIEDG-IFEVKSTSGDTHLGGEDFDNRMVNHFTQEFK
                              ***********:***::* :***:**:****************** ****
  NLS
TRINITY_DN156808_c2_g1_1      RKYGKDISKNNRSIRRLRTACERAKRTLSSSTEASIEIDSLFDGVDFYTK
Mya_contig00268               RKHKKDLSTNKRALRRLRTACERAKRTLSSSTQASIEIDSLFEGVDYYTS
                              **:  **:*.*:*::*****************:*********:***:**.
  Motif V
TRINITY_DN156808_c2_g1_1      VSRARFEELCGDLFRLTMEPVEKALRDAKMDKSKINDVVLVGGSTRIPRI
Mya_contig00268               ITRARFEELNADLFRGTLEPVEKALRDAKFDKASMNDIVLVGGSTRIPKI
                              ::******* .**** *:***********:**:.:**:**********:*
                                    N-X-S                              A
TRINITY_DN156808_c2_g1_1      QKLLKDFMNGKELNKSINPDEAVAYGAAVQAAILSGDQSDAIKDVLLVDV
Mya_contig00268               QKLLQDFFNGKELNKSINPDEAVAYGAAVQAAILHGDKSEEVQDLLLLDV
                              ****:**:************************** **:*:  ::*:**:**
TRINITY_DN156808_c2_g1_1      TPLSLGIETAGGVMTKIIERNTKIPTKATQTFTTYSDNQPAVNIQVYEGE
Mya_contig00268               TPLSLGIETAGGVMTSLIKRNTTIPTKQTQTFTTYSDNQPGVLIQVYEGE
                              ***************.:*:***.**** ************.* *******
TRINITY_DN156808_c2_g1_1      RAMTKDNNPLGRFDLIGIPPAPRGVPQIEVTFDIDANGIMNVSAEDKSTK
Mya_contig00268               RAMTKDNNLLGKFELTGIPPAPRGVPQIEVTFDIDANGILNVTAADKSTG
                              ******** **:*:* ***********************:**:* ****
TRINITY_DN156808_c2_g1_1      KRNNITITNDSGRLSKADIERMVNDADKYREEDEKQRQRIEARNKLENYM
Mya_contig00268               KENKITITNDKGRLSKDEIDRMVNDAEKYKAEDETQRERITAKNSLESYS
                              *.*:******.***** :*:******:**: ***.**:** *:*.**.*
TRINITY_DN156808_c2_g1_1      FSVKQAVSEAD-DSKVSAEDKTKVTELCDECMKWMDSNSLADKDEFEHRL
Mya_contig00268               FNMKSTVEDENLKDKISAEDKKTIIDKCNDVISWLDSNQQGEKDEFEYKQ
                              *.:*.:*.: : ..*:*****..: : *:: :.*:***. .:*****::
TRINITY_DN156808_c2_g1_1      DDLQKQCSPVMVKLHGGDRQQGQPG----------SGPAPGGQPAGG--H
Mya_contig00268               KELEAVCNPIVTKLYQGGAAPGGAGGMPGNFGAGAGGAPPGAAPGGGSGS
                              .:*:  *.*::.**: *.   * .*          .*..**. *.**
TRINITY_DN156808_c2_g1_1      QGPTVEEMD
Mya_contig00268               GGPTIEEVD
                               ***:**:*
```

Supplementary Figure S3. Comparison of two hsp70 transcripts, one identified in this study and one identified in Sleight *et al.* (2016) annotated with domain motifs. Domain motifs are annotated in red with the following characteristic signature motifs as described in Rensing & Maier (1994) unless stated: I: [IVL]-D-[LF]-G-T-T-x-S' II: D-[LF]-G(3)-T-F-D, III: [TS]-[VC]-P-A-[YN]-[FY]-N, IV: [NP]-[EG]-P-[TS]-A-A and V: R-A-[RK]-F-E-[ED]-[LM]. Also annotated: A = the hydrophobic linker site between the nuclear binding domain (NBD) and the substrate binding domain (SBD) (Zuiderweg *et al.* 2013) and two N-linked glycosylation sites (NKSI and NVSA (consensus of N-X-S)), the second one of which is imperfect in Contig00268 (Munro & Pelham, 1986; Laursen *et al.* 1997). The putative nuclear localisation signal domain (KRK**.KDL***.RAL*R) is marked (NLS), although DN156808 may not have sufficient positively charged amino acids in this region to satisfy this motif. The terminal motif of EEVD denotes a cytoplasmic location.

References:

Haas Bj, Papanicolaou A, Yassour M. *et al.* (2013) De novo transcript sequence reconstruction from RNA-Seq: reference generation and analysis with Trinity. Nature protocols, **8**, 10.1038/nprot.2013.1084.

Laursen JR, di Liu H, Wu X-J, Yoshino T. P. (1997) Heat-shock response in a molluscan cell line: Characterisation of the response and cloning of an inducible HSP70 cDNA. Journal of Invertebrate Pathology 70, 226-33.

Munro S & Pelham HRB. (1986) An Hsp70-like protein in the ER: Identity with the 78 kd glucose-regulated protein and immunoglobulin heavy chain binding protein. Cell 46, 291-300.

Rensing SA & Maier U-G (1994) Phylogenetic analysis of the stress-70 protein family. Journal of Molecular Evolution. 38, 80-86.

Zuiderweg ERP, Bertelsen EB, Rousaki A, Mayer MP, Gestwicki JE, Ahmad A. (2013) Allostery in the Hsp70 chaperone proteins. Topics in Current Chemistry, **328**, 99-153.
